# TRPA1-kinase axis polarization: Nepetalactone drives pest repulsion and predator attraction via divergent PKC/CaMKII signaling

**DOI:** 10.1016/j.jare.2025.07.026

**Published:** 2025-07-17

**Authors:** Jianying Li, Bo Wang, Yilin Wang, Fen Li, Zhen Li, Xiaoxia Liu, Songdou Zhang

**Affiliations:** a Plant Protection Research Institute, Guangdong Academy of Agricultural Sciences, Key Laboratory of Green Prevention and Control on Fruits and Vegetables in South China Ministry of Agriculture and Rural Affairs Guangdong Provincial Key Laboratory of High Technology for Plant Protection, Guangzhou 510640, China; b Department of Entomology and MOA Key Lab of Pest Monitoring and Green Management, College of Plant Protection, China Agricultural University, Beijing 100193, China; c Sanya Nanfan Research Institute, Hainan University, Yazhou, Sanya 572024, China

**Keywords:** Nepetalactone, TRPA1, Push-pull strategy, Condylognatha pests, Kinase signaling, Chemoperception

## Abstract

**Introduction:**

Nepetalactone, an iridoid compound derived from *Nepeta cataria*, is a potent TRPA1 agonist known to repel hematophagous insects. However, its ecological roles and molecular mechanisms within agricultural ecosystems remain largely unexplored.

**Objectives:**

This study aimed to investigate: (1) the species-specific effects of nepetalactone on pests and predator insects, (2) its action on TRPA1 orthologs, and (3) its potential for push–pull pest control strategies.

**Methods:**

We employed multi-tiered approaches including behavioral assays (two-choice tests, Y-tube olfactometry), molecular techniques (RNAi, qRT-PCR, FISH, Western blot), calcium imaging, and electrophysiology to assess behavioral responses and underlying mechanisms. Field trials further validated pest suppression efficacy.

**Results:**

Nepetalactone exhibits dual functionality: it activates TRPA1 orthologs through conserved cysteine-rich domains, triggering calcium influx and aversion in three sap-sucking pests (*Cacopsylla chinensis*, *Diaphorina citri*, *Megalurothrips usitatus*; Order: Hemiptera). Conversely, it paradoxically attracts two predatory ladybeetles (*Harmonia axyridis*, *Propylea japonica*). Mechanistically, this behavioral dichotomy arises form divergent downstream kinase signaling – PKCα mediates repellency in pests, whereas CaMKII drives attraction in predators – despite conserved TRPA1 activation. Field trails validated operational synergy: nepetalactone combined with predatory ladybeetles achieving >90 % pest suppression through integrated push–pull effects. Our integrated approach (calcium imaging, RNAi, electrophysiology) reveals that TRPA1-kinase axis polarization governs species-specific chemoperception.

**Conclusion:**

These findings resolve the ecological paradox wherein a single semiochemical elicits opposing trophic behaviors. nepetalactone is established as a keystone agent for sustainable pest management, providing a molecular blueprint for engineering TRPA1-targeted repellents. Furthermore, it advances push–pull strategies by exploiting predator-pest signaling networks.

## Highlights

• Nepetalactone activates TRPA1 to repel hemipteran pests but attract predatory ladybeetles.
• A conserved CVT motif in TRPA1 orthologs mediates nepetalactone-induced aversion in pest.
• TRPA1 activation drives pest repulsion via PKCα but predator attraction via CaMKII kinase.
• Combining nepetalactone with ladybeetles achieves >90 % pest suppression, demonstrating operational efficacy.

## Graphical abstract

**Figure:**
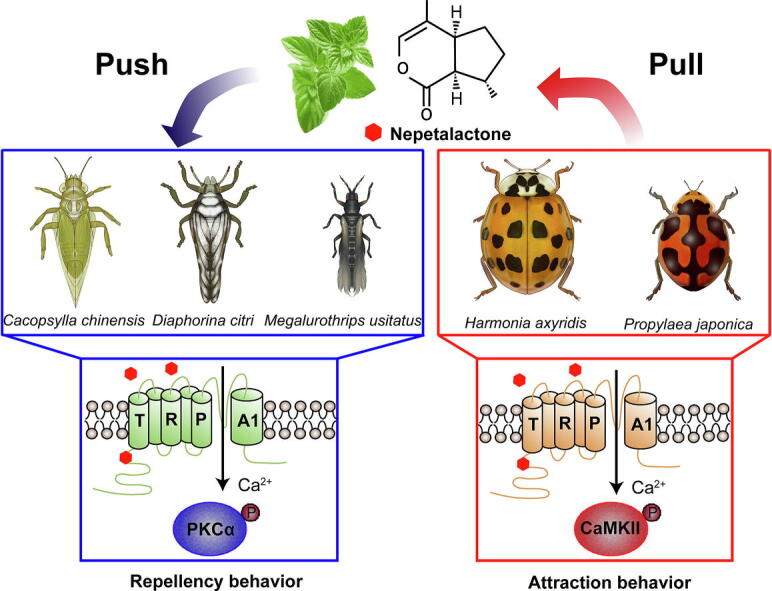

## Introduction

Sap-sucking pests within the Condylognatha superorder, particularly Thysanoptera (thrips) and Hemiptera species (psyllids, whiteflies, and others), pose persistent threats to global agriculture through phloem feeding and rapid reproduction [[1], [2], [3]]. Overreliance on chemical controls has exacerbated insecticide resistance and food safety concerns [[4], [5], [6]], necessitating sustainable alternatives. While natural predatory enemies like *Harmonia axyridis* and *Propylaea japonica*, demonstrate effective biocontrol potential on Condylognatha pests [[7], [8], [9]], their mass rearing costs and field efficiency limitations highlight the need for optimized biological strategies.

Push-pull systems employing plant-derived volatiles offer an eco-friendly solution by simultaneously repelling pests and attracting natural enemies [[10], [11], [12], [13]]. Notably, nepetalactone – the principal iridoid in catnip which scientifically known as *Nepeta cataria* – exhibits dual ecological functions: a potent insect repellent exceeding DEET (N, N-Diethyl-3-methylbenzoyl amide) efficacy [[14], [15], [16]] and a key aphid sex pheromone component attracting aphidophagous predators like *Chrysopa septempunctata* [17,18]. Recent studies confirm its broad-spectrum repellency against hematophagous insects [19,20], yet its application potential against phytophagous Condylognatha pests and differential effects on pests versus predators remain unclear.

Nepetalactone exhibits species-specific neuromodulatory effects through distinct molecular targets. While activating μ-opioid receptors in felines to induce characteristic behavioral responses [16], it primarily engages the chemosensory TRPA1 channel in insects [19]. Notably, while *N. cataria* and nepetalactone do not activate human TRPA1, this conserved TRP (transient receptor potential) channel super-family member exhibits broad phylogenetic distribution across insects and mammals [21,22]. This evolutionary divergence is particularly intriguing given TRPA1′s conserved role as a multimodal detector of noxious stimuli across metazoans [23], yet its structural complexity in insects suggests adaptive specialization. Insect TRPA1 channels display expanded genetic diversity compared to vertebrates, featuring variant-specific chemical sensitivities potentially shaped by plant-insect coevolution [19]. Structurally, these non-selective cation channels integrate multiple activation modules – ankyrin repeats for mechanical sensing, redox-sensitive cysteine residues, and enhancer domains for thermal detection [24] – creating versatile signaling platform. As a Ca^2+^-permeable cationic channel, TRPA1′s functional dichotomy emerges through divergent downstream signaling: Protein kinase C (PKC)-dependent pathways regulate cold adaptation in *Caenorhabditis elegans* [25], while Ca^2+^/calmodulin-dependent protein kinase (CaMK) mediates cardiac modulation in mammals [26]. This raises critical questions about TRPA1′s signaling partners in mediating nepetalactone-driven behaviors – could conserved channel architecture couple with lineage-specific effectors to produce opposing ecological responses?

To address this knowledge gap, we investigate TRPA1-mediated nepetalactone responses across three Condylognatha pests (*Cacopsylla chinensis*, *Diaphorina citri*, and *Megalurothrips usitatus*) and two natural predatory enemies (*H. axyridis*, *P. japonica*). Our study aims to: (1) Characterize species-specific behavioral responses to plant-derived nepetalactone; (2) Determine TRPA1 ortholog conservation and functional divergence; (3) Elucidate differential engagement of PKC/CaMK signaling pathways; (4) Evaluate field applicability of nepetalactone-based push–pull strategies. By resolving how a single phytochemical achieves pest deterrence and predator recruitment through TRPA1 plasticity, this work advances both chemosensory biology and sustainable agriculture. Our findings establish an evolutionary framework for designing targeted pest management strategies that exploit sensory channel diversification.

## Materials and methods

### Insect colonies and rearing conditions

Three sap-sucking pest species were maintained under controlled laboratory conditions: (1) *C. chinensis* (Hemiptera: Psyllidae): Originally collected from pear orchards in Daxing, Beijing, China (39°26′N, 116°13′E; September 2018), reared on *Pyrus sinkiangensis* (Korla fragrant pear) at 25 ± 1 °C, 65 ± 5 % RH, and 12L:12D photoperiod at China Agricultural University (Beijing, China) [27]. (2) *D. citri* (Hemiptera: Liviidae): Sourced from citrus orchards in Zhanjiang, Guangdong, China (20°35′N, 109°31′E; July 2017) maintained on healthy *Citrus limon* (L.) Osbeck plants at 26 ± 1°C, 65 ± 5 % RH, and 14L:10D photoperiod [28]. (3) *M. usitatus* (Thysanoptera: Thripidae): Collected from cowpea (*Vigna unguiculata*) field in Sanya, Hainan, China (18°14′N, 109°31′E), reared on cowpea pods/cowpea seedlings under identical conditions to *D. citri*.

Predatory beetles *H. axyridis* and *P. japonica* (Coleoptera: Coccinellidae) were obtained from pear orchards in Fangshan, Beijing, China (39°30′N, 115°25′E; September 2021) and maintained on *Acyrthosiphon pisum* colonies (hosted on *Vicia faba*) at 26 ± 1°C, 65 ± 5 % RH, and 14L:10D photoperiod [29,30].

### Two-way choice behavioral assays

For *C. chinensis* and *D. citri*, tested in cylindrical plastic chambers (10 cm in diameter and 15 cm in height) with ventilation ports as described in *Eupeodes corollae* [31]. Two equally sized plant leaves were inserted into 1.5 mL centrifuge tubes – one treated with nepetalactone (10 μM–10 mM in paraffin oil; MedChemExpress HY-129434A) and the other with paraffin oil control (10 μL each). For *M. usitatus*, evaluated in plastic petri dishes with a diameter of 9 cm containing nepetalactone-treated vs. control cowpea pod segments (1 cm^2^). After 6 h starvation, 10–20 insects (nymphs/adults) or 10 predators were introduced centrally. The preference index (PI) was calculated as (T − C)/(T + C) after 15 min, where T and C represent counts on treatment and control sides, respectively.

### Y-shaped tube olfactometry and Electroantennography (EAG)

A glass Y-shaped tube (2.5 cm inner diameter, 10 cm arm length) was purged with activated charcoal-filtered air (300 ml/min) [31]. Tested arm received filter papers impregnated with nepetalactone (10 μM, 100 μM, 1 mM) or paraffin oil (20 μL). Individual starved adults were introduced to the trunk, with choices recorded within 10 min. To eliminate positional bias, the apparatus was rotated every three replicates and ethanol-sterilized (180 °C for 3 h) after 10 trials. All tests (n ≥ 30 per group) were conducted between 8:00 and 17:00 under 1000 lx illumination.

Antennal preparations from cold-anesthetized *H. axyridis* females were mounted between glass capillary electrodes containing 0.1 M KCl. Odor stimuli were delivered via a Pasteur pipette (2 mm distance) containing filter paper impregnated with 100 μM nepetalactone (10 μL in paraffin oil). Signals were amplified through a Syntech 103AC/DC preamplifier and recorded using IDAC-4-USB with EAG Pro v2.7 software (Syntech, Germany). Stimulus parameters: 30 mL/s baseline airflow, 0.2 s pulses (10 mL/s) at 30 s intervals. Each antenna received sequential paraffin oil (control) and nepetalactone stimuli (n = 6 biological replicates).

### Sequence analysis of TRPA1 orthologs

Full-length TRPA1 orthologs were retrieved from transcriptome database or NCBI (Accessions: PP096834 for *CcTRPA1*, PP096835 for *DcTRPA1*, PP096836 for *MuTRPA1*, XM_045607606.1 for *HaTRPA1*, and PP096837 for *PjTRPA1*) [23,31]. Tertiary protein structures were predicted using AlphaFold2 and validated via PROCHECK Ramachandran plot in SAVES 6.0 [32]. Molecular docking simulations (AutoDock 4.2.6) identified nepetalactone binding pocket, with optimal models selected based on binding affinity (kcal/mol). Domain architecture was analyzed using SMART, and multiple amino acid sequence alignments were analyzed using DNAman 9.0 software. The phylogenetic tree was constructed using MEGA10.1.8 software (neighbor-joining method).

### Calcium imaging and fluorescence assays

TRPA1 ORFs were cloned into pcDNA3.1(+)-mCherry vectors and transfected into HEK293T cells using StarFect Lip2000 Transfection Reagent (Cat#C105, GenStar, China) [26]. Intracellular Ca^2+^ flux was monitored via Fluo-4 AM (Cat# S1061, Beyotime, China) using Leica SP8 confocal microscopy (Weztlar, Germany) and the MD i3x microplate reader (San Jose, USA). Relative fluorescence changes were quantified as ΔF/F0, where F0 represents baseline fluorescence and ΔF denotes stimulus-induced variation. To analyze chimeras, reverse PCR in combination with homologous recombination was utilized to replace the cysteine-rich linker domain (Cys. domain) of CcTRPA1, DcTRPA1, MuTRPA1, and HsTRPA1 (pCMV-TRPA1 human-3 × FLAG, P35762, MiaoLingBio, China) with primers in Table S1. Molecular docking-guided site-directed mutagenesis was conducted using Mut Express II Fast Mutagenesis Kit V2 (Cat#C214, Vazyme, China), with mutations validated by Sanger sequencing.

For kinase co-transfection studies, HEK293T cells in 96-well plates were co-transfected with either pest system (CcTRPA1 + CcPKCα) or predator system (HaTRPA1 + HaCaMKII) for 0.125 μg plasmid per gene construct. At 48 h post-transfection, calcium flux kinetics was recorded using a BioTek Synergy H1 multimode microplate reader (Agilent, USA) under standardized protocol: (1) baseline stabilization (0–30 s); (2) automated nepetalactone injection (100 μM final); (3) continuous monitoring across 40–350 s. Background-subtracted ΔF/F0 values were integrated as area-under-curve (AUC) using GraphPad Prism 8.0.

### Gene expression analysis by qPCR and fluorescence in situ Hybridization

Adult females of all three species were collected at 1, 3, 5, 7, and 9 days post-eclosion for developmental stage analysis. To assess nepetalactone and inhibitor effects (AP-18: Cat# HY-W014421; HC-030031: Cat# HY-15064; MedChemExpress, China), cohorts of at least twenty females were collected at 0.5, 1, 3, and 6 h post-treatment. Total RNA extraction, cDNA synthesis, and qRT-PCR analysis were performed using the TaKaRa RNA Extraction Kit (Cat# 9767, Japan), Takara PrimeScript RT reagent kit (RR047A, Takara, Japan), and Takara TB Green Premix Ex Taq II kit (RR820A, Takara, Japan) according to the provided instructions, respectively. Reactions were normalized to *β-actin* and quantified using the 2^−ΔΔCT^ method [33].

For FISH experiments, Cy3-labeled TRPA1 probes (GenePharma, Shanghai, China) were hybridized to Carnoy's-fixed specimens following the previously described protocol [26]. Briefly: (1) **Fixation**: 24 h in Carnoy's solution; (2) **Decolorization**: 72 h in 6 % hydrogen peroxide/ethanol; (3) **Hybridization**: 24 h at 42 °C with 5 uM fluorescent probes in hybridization buffer (20 mM Tris-HCl, pH 8.0; 0.9 M NaCl; 0.01 % sodium dodecyl sulfate; 30 % formamide); (4) **Washing**: 3 × 10-min PBS rinses; (4) **Counterstaining**: DAPI (1 μg/mL, Solarbio, Beijing, China) at room temperature for 15 min. Images were acquired using a Leica SP8 fluorescence microscope (Weztlar, Germany).

### Single Sensillum Recordings (SSR)

Extracellular electrophysiological recordings targeted TRPA1-expressing trichoid sensilla in three pest species. Sexually mature females were prepared as follows: (1) *C. chinensis* and *D. citri*: head-immobilized in pipette tips with reference electrodes inserted into compound eyes following the established procedure in *D. melanogaster* [34]; (2) *M. usitatus*: abdominally secured on glass slides using double-sided tape (abdominal reference electrode). A tungsten microelectrode (IDAC-4 amplifier, Syntech) was positioned at sensillum bases under Leica Z16 APO microscope. Stimuli consisted of 100 μM nepetalactone (30 μL on filter paper) delivered via CS-55 controller (300 ms pulse, 30 mL/s airflow). Neural responses were quantified as peak frequency differences (1 s pre- vs post-stimulus) using Autospike 32 software (Syntech, Buchenbach, Germany).

### TRPA1 knockdown and pharmacological inhibition

Species-specific dsRNAs (1000 ng/μL) were synthesized using T7 promoter primers (Table S1) and delivered via: (1) *C. chinensis* and *D. citri*: stem-leaf feeding device [26]; (2) *M. usitatus*: artificial diet (20 % sucrose + 500  ng/μL dsRNA; (3) *H. axyridis*/*P. japonica*: dorsal abdominal microinjection (Nanoject II, Drummond Scientific Company). TRPA1 inhibitors (AP-18/HC-030031) were dissolved in 0.1 % DMSO vehicle using analogous delivery methods as dsRNA. The optimum efficiency of RNAi and inhibitor treatment was assessed by collecting individuals at different time points post-treatment for qRT-PCR analysis. For behavioral experiments and single sensillum recordings, female adults were selected approximately 2 days after RNAi or inhibitor treatment.

### Subcellular localization and Western blot

TRPA1 and kinase ORFs (CcTRPA1-Flag, DcTRPA1-Flag, MuTRPA1-Flag, HaTRPA1-Flag, PjTRPA1-Flag, CcPKCα-His, CcCaMKII-HA, HaPKCα-His, HaCaMKII-HA) were cloned into pCDNA3.1 vector via homologous recombination. HEK293T cells in 24-well plates were transfected with 1 μg plasmid DNA. At 48 h post-transfection, membrane/cytoplasmic fractions were isolated (Beyotime membrane protein kit, Cat#P0033, China). Proteins were resolved on 8 % (TRPA1) or 10 % (β-actin) SDS-PAGE gels and transferred to PVDF membranes (120 V, 60 min). Membranes were blocked with 5 % skimmed milk, probed with anti-Flag mAb (Abclonal, Cat#AE005, 1:3000) or β-actin mAb (TransGen, Cat#HC201, 1:5000), and detected via HRP-ECL (Bio-Rad Clarity, USA).

For Co-immunoprecipitation (Co-IP) and phosphorylation, HEK293T cells co-transfected with TRPA1/kinase constructs (0.3 μg each) were treated with 100 μM nepetalactone or EGTA for 10 min. Lysates (Beyotime IP buffer Cat#P0013 with protease/phosphatase inhibitor) were incubated with anti-tag magnetic beads (Abclonal Cat#AE092/AE086/AE105; Beyotime P2108) at 4 °C for 1 h. Input and IP fractions were analyzed by WB using Mouse mAbs (1:3000, Cat#AE005/AE003/AE008, Abclonal) with 5 % skim milk blocking. For phosphorylation detection, 5 % BSA was used for blocking and antibody dilution, with pan Phospho-Serine/Threonine Mouse mAb (1:1000, Cat#AP1067, Abclonal) and Goat Anti-Mouse IgG (H + L) (1:3000).

### Field caging assay

The field cages (180 × 180 × 200 cm) contained central host plants (*Pyrus* spp., *Citrus limon*, or *Vigna unguiculata*) treated with *N. cataria* extract (5 mg/ml) or sterile water. Pest populations (*C. chinensis*: 50 adults; *D. citri*: 30 adults, *M. usitatus*: 100 adults) and predators (*H. axyridis*/*P. japonica*: 10 females) were introduced separately. Survival rates were counted after 24 h (n = 9 replicates).

### Statistical analysis

Data (mean ± standard error, SEM) were analyzed in GraphPad Prism 8.0 software. In the Y-shaped tube assay, differences were examined using the chi-square goodness-of-fit test (*p < 0.05. **p < 0.01, ***p < 0.001, ns: not significant). Student's *t*-test was employed for analyzing two groups of data (*p < 0.05, **p < 0.01, ***p < 0.001, ns: not significant). For multiple comparisons, one-way ANOVA was conducted, followed by Tukey's HSD test (different letters indicate *p* < 0.05).

## Results

### Three Condylognatha pest species exhibit concentration-dependent aversion to plant-derived nepetalactone

**Fig. 1:**
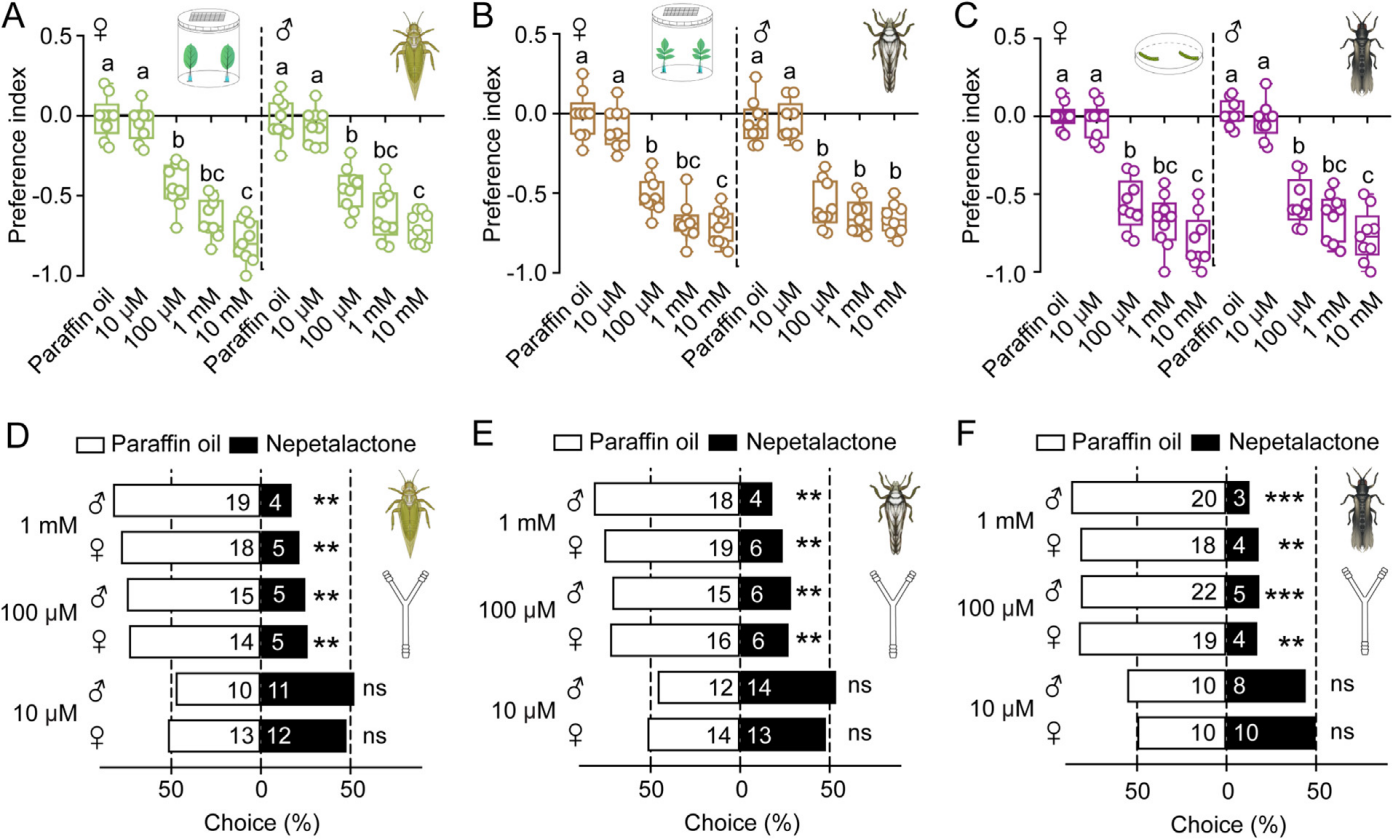
**Nepetalactone elicits dose-dependent aversion in Condylognatha pests.** A–C: Two-way choice assays assessing repellency in *C. chinensis*, *D. citri*, or *M. usitatus adults*, respectively. Preference index (PI = [T-C)/[T + C) for nepetalactone (10 μM–10 mM) vs. paraffin oil control (mean ± SEM, n = 9 biological replicates; 12–20 insects/replicate). Different lowercase letters indicate significant differences (ANOVA, Turkey’s HSP; *p* < 0.05). D–F: Y-shaped tube assays confirming the repellency in *C. chinensis*, *D. citri*, or *M. usitatus*, separately. Each group comprised three independent biological replicates with a total of approximately 30–35 adults. Statistically significant differences were evaluated using the chi-square test in SPSS 20.0 software (ns, not significant; **, *p* < 0.01; ***, *p* < 0.001).

To evaluate behavioral responses of three Condylognatha pests (*C. chinensis*, *D. citri*, and *M. usitatus*) to nepetalactone, we conducted two-choice assays to assess their sensitivity under standardized conditions. Adult *C. chinensis* or *D. citri* individuals were placed in round plastic containers with paired stimuli: fresh leaves sprayed with nepetalactone versus paraffin oil controls. For *M. usitatus* adults, assays were conducted in petri dishes containing two bean substrates. The preference index (PI), calculated as (T − C)/(T + C) where T and C represent treatment and control values, quantified odor source selectively. Dose-response analyses revealed significant nepetalactone aversion across all species (Fig. 1A–C). In *C. chinensis*, females exhibited PIs of −0.05 ± 0.11, −0.43 ± 0.14, −0.64 ± 0.12, and −0.79 ± 0.13 at 10 μM, 100 μM, 1 mM, and 10 mM nepetalactone, respectively, while males showed PIs of −0.07 ± 0.11, −0.46 ± 0.13, −0.61 ± 0.16, and −0.70 ± 0.10 (Fig. 1A). Similar dose-dependent trends were observed in *D. citri* (females: −0.09 ± 0.13, −0.51 ± 0.11, −0.68 ± 0.12, and −0.71 ± 0.11; males: −0.05 ± 0.12, −0.57 ± 0.15, −0.64 ± 0.11, and −0.66 ± 0.10) (Fig. 1B) and *M. usitatus* (females: −0.03 ± 0.12, −0.57 ± 0.16, −0.68 ± 0.17, and −0.80 ± 0.17; males: −0.03 ± 0.12, −0.55 ± 0.15, −0.65 ± 0.15, and −0.77 ± 0.17) (Fig. 1C). Statistical significance was particularly pronounced at ≥100 μM concentrations in both female and male adults of the three species. Specifically, *p* = 5.42E-06, 1.18E-08, 1.13E-09 for females and 3.95E-06, 3.35E-07, 1.26E-09 for males when using concentrations of 100 μM, 1 nM, and 10 mM in *C. chinensis*; 4E-07, 1.43E-08, 4E-09 for females and 9E-07, 2.53E-08, 9E-09 for males in *D. citri*; 7.74E-08, 1.08E-08, 6.83E-10 for females and 2.67E-08, 3.65E-09, 9.07E-10 for males in *M. usitatus*, respectively (Fig. 1A–C). Complementary Y-tube assays corroborated these findings, demonstrating consistent aversion at ecologically relevant concentrations of 100 μM and 1 mM (Fig. 1D–F).

Notably, nymphal stages of all three species displayed enhanced sensitivity compared to adults, exhibiting significant aversion even at sub-100 μM concentrations (1–100 μM) (Fig. S1A–C). These findings provide further confirmation that both nymphs and adults of three Condylgnatha species displayed strong aversion behavior towards plant-derived nepetalactone.

### Nepetalactone activates TRPA1 orthologs of three sap-sucking pests *in vitro*

**Fig. 2:**
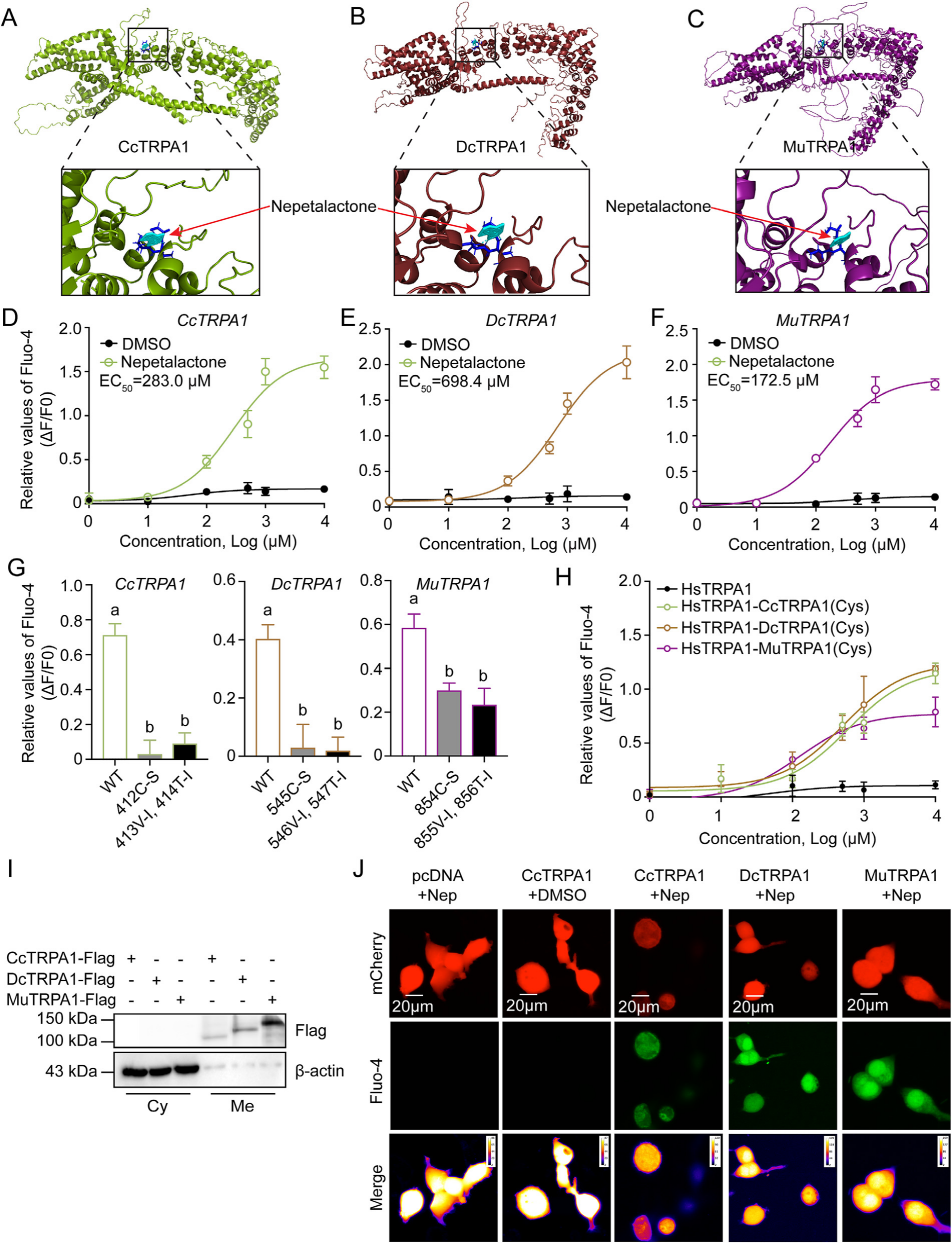
**TRPA1 mediates nepetalactone responses in Condylognatha pests.** A–C: Predicted tertiary protein structures of pest TRPA1 variants with nepetalactone docking (red arrows: binding sites). D–F: Dose-dependent calcium flux in TRPA1-expressing HEK293T cells. To build the recombinant vectors, the full-length coding sequence of each TRPA1 variants was inserted into the pcDNA3.1(+)-mCherry vector, respectively. DMSO treatment was examined as the negative control (EC_50_ values shown; mean ± SEM, n = 3). G: Mutation of binding sites in TRPA1 orthologs markedly decreased nepetalactone sensitivity. *CcTRPA1* mutations included 412C-S (cysteine 412 changed to serine), 413V-I (valine 413 changed to isoleucine), and 414T-I (threonine 414 changed to isoleucine). *DcTRPA1* mutations included 545C-S (cysteine 545 changed to serine), 546V-I (valine 546 changed to isoleucine), and 547V-I (threonine 547 changed to isoleucine). *MuTRPA1* mutations included 854C-S (cysteine 854 changed to serine), 855V-I (valine 855 changed to isoleucine), and 856T-I (threonine 856 changed to isoleucine). Mean values ± SEM; different lowercase letters: ANOVA, *p* < 0.05. H: Chimeric human TRPA1 with pest linker domains restores responsiveness. I: Membrane localization of TRPA1 variants (Cy: Cytoplasm; Me: Membrane). J: Representative images of Fluo-4 Ca^2+^ imaging (mCherry: transfection marker; Fluo-4: Ca^2+^; Merge: merged imaging of mCherry and Fluo-4 signals). Scale bar: 20 μm. (For interpretation of the references to colour in this figure legend, the reader is referred to the web version of this article.)

While TRPA1 is known to mediate nepetalactone aversion in dipteran models (e.g., flies and mosquitoes), its role in hemipteran pests remains unexplored. We cloned full-length TRPA1 orthologs from three sap-sucking pests (*C. chinensis CcTRPA1*, *D. citri DcTRPA1*, and *M. usitatus MuTRPA1*) using species-specific primers (Table S1). Sequence analysis revealed conserved six-transmembrane architectures across all orthologs, with distinct structural variations: *CcTRPA1* (952 aa), *DcTRPA1* (1073 aa), *MuTRPA1* (1403 aa) exhibited 10, 14, and 16 N-terminal ankyrin repeats, respectively – a feature potentially influencing ligand specificity (Figs. 2A–C and S2). Molecular docking identified a conserved CVT motif (Cysteine-Valine-Threonine) critical for nepetalactone binding, with evolutionary analysis showing striking conservation across 10 insect species versus absence in mammals (CPI motif) (Fig. S3A). Phylogenetic reconstruction confirmed expected evolutionary relationships: *CcTRPA1* and *DcTRPA1* clustered with Hemiptera, while *MuTRPA1* aligned with Thysanoptera, all diverging markedly from coleopteran predators and mammals (Fig. S3B). Developmental profiling demonstrated progressive upregulation of all three *TRPA1* orthologs during female adult maturation (days 1–9 post-eclosion) (Fig. S3C–E), correlating with behavioral aversion development.

Functional validation via calcium mobilization assays confirmed dose-dependent TRPA1 activation (EC_50_: CcTRPA1 = 283.0 μM; DcTRPA1 = 698.4 μM; MuTRPA1 = 172.5 μM) (Fig. 2D–F). Site-directed mutagenesis of the CVT motif (C or VT) abolished nepetalactone responsiveness (Δfluorescence was obvious reduction vs. wild-type) (Figs. S4A–C and 2G). Crucially, chimeric studies demonstrated functional transferability: grafting the cysteine-rich linker from pest TRPA1 orthologs conferred nepetalactone sensitivity to human *TRPA1* (Fig. 2H), whereas reciprocal substitutions eliminated responsiveness (Fig. S4D). Membrane localization of all three TRPA1 orthologs (Fig. 2I) and nepetalactone-induced calcium flux (Fig. 2J) further corroborated their receptor functionality. These findings conclusively establish TRPA1 as the direct molecular target of nepetalactone in sap-sucking pests.

### TRPA1 orthologs mediate nepetalactone-induced repellency in three sap-sucking pests

**Fig. 3:**
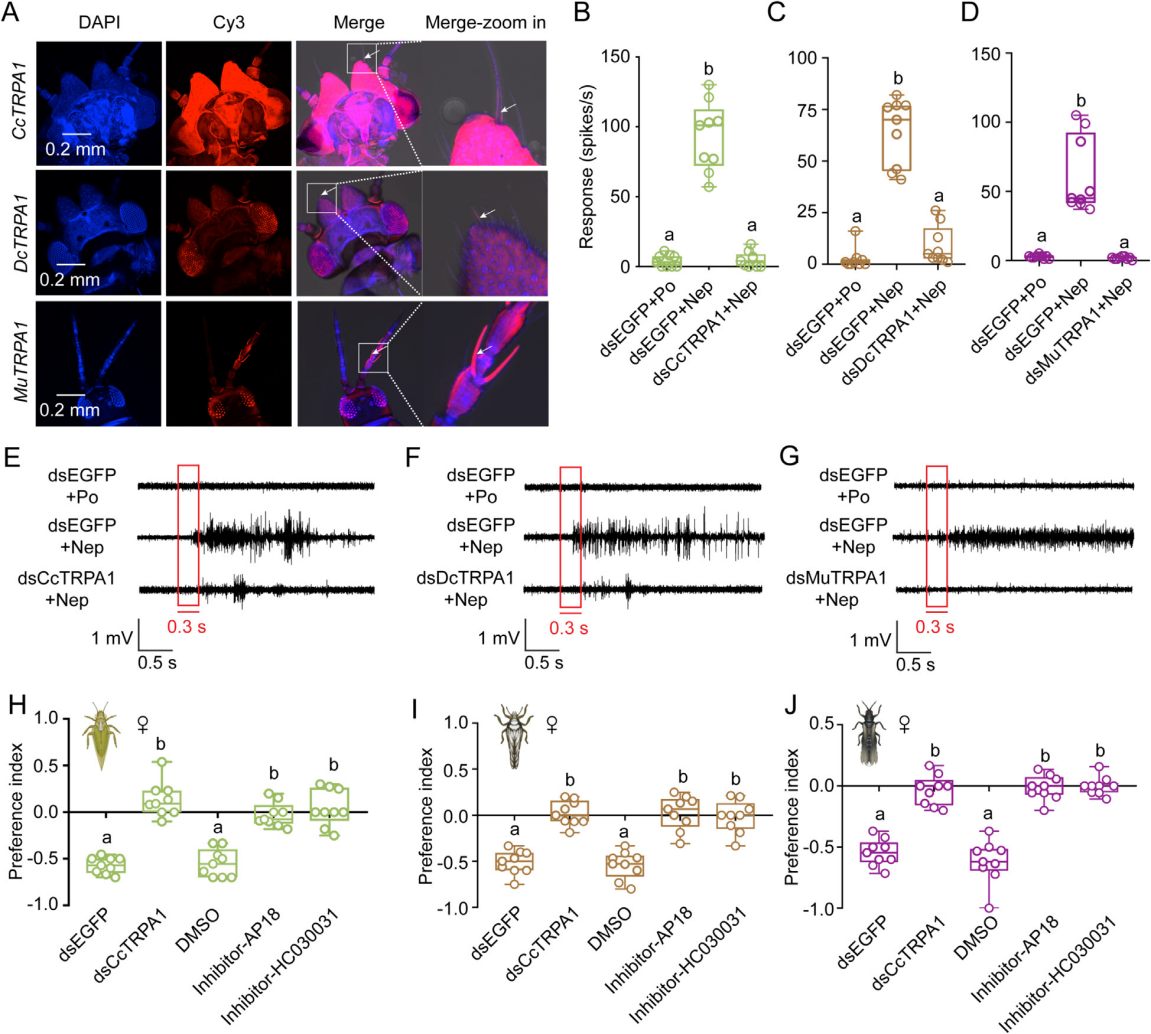
**TRPA1 mediates nepetalactone aversion via antennal sensilla.** A: FISH localization of TRPA1 transcripts in pest heads (arrows: trichoid sensilla). Scale bar: 0.2 mm. DAPI: cell nuclei stained with DAPI and visualized in blue. Cy3: TRPA1 signal labeled with Cy3 and visualized in red. Merge: merged imaging of BF, DAPI, and TRPA1-Cy3 signals. B-D: Single-sensillum recording (SSR) response values of after TRPA1 knockdown in response to 100 μM nepetalactone. Adults treated with “dsEGFP + paraffin oil” or “dsEGFP + nepetalactone” served as negative and positive controls, separately. E–G: Representative SSR traces. H–J: TRPA1 knockdown/inhibition abolishes repellency. The results in Figure B–D and H–J are shown as mean values ± SEM with nine independent biological replicates (Different lowercase letters: ANOVA, *p* < 0.05). (For interpretation of the references to colour in this figure legend, the reader is referred to the web version of this article.)

To establish the functional role of TRPA1 orthologs in mediating nepetalactone aversion *in vivo*, we integrated electrophysiological, genetic, pharmacological, and behavioral approaches across *C. chinensis*, *D. citri*, and *M. usitatus*. Transcriptional profiling revealed rapid upregulation of *CcTRPA1*, *DcTRPA1*, and *MuTRPA1* within 1–6 h after nepetalactone exposure (Fig. S5A–C), with tissue-specific qRT-PCR showing predominant expression in the head region (Fig. S6A–C). Fluorescence *in situ* Hybridization (FISH) localized all three orthologs to antennae trichoid sensillas as indicated by white arrow (Fig. 3A), suggesting a chemosensory role. Single-sensillum recordings demonstrated concentration-dependent neuronal activation by nepetalactone in wild-type individuals (Fig. 3B–G). RNAi-mediated successful knockdown of TRPA1 abolished this response (Fig. S7A–C), with dsTRPA1-treated sensilla showing markedly reduced firing rates compared to dsEGFP controls (Fig. 3B–G). Paraffin oil-treated negative controls exhibited baseline activity, confirming stimulus specificity.

Pharmacological inhibition further validated TRPA1 dependency: AP18 and HC030031 (TRPA1 antagonists) suppressed both *TRPA1* transcription and intracellular Ca^2+^ flux in a dose-dependent pattern (Fig. S7D–I). Crucially, behavioral assays revealed complete loss of nepetalactone aversion in inhibitor-treated and RNAi groups, mirroring the null response to paraffin oil (Fig. 3H–J). These multimodal evidences establish TRPA1 as the principal mediator of nepetalactone repellency in three sap-sucking pests, with *in vivo* functionality conserved across divergent hemipteran lineages.

### CcTRPA1-CcPKCα axis mediates nepetalactone-induced repellency in *C. chinensis*

**Fig. 4:**
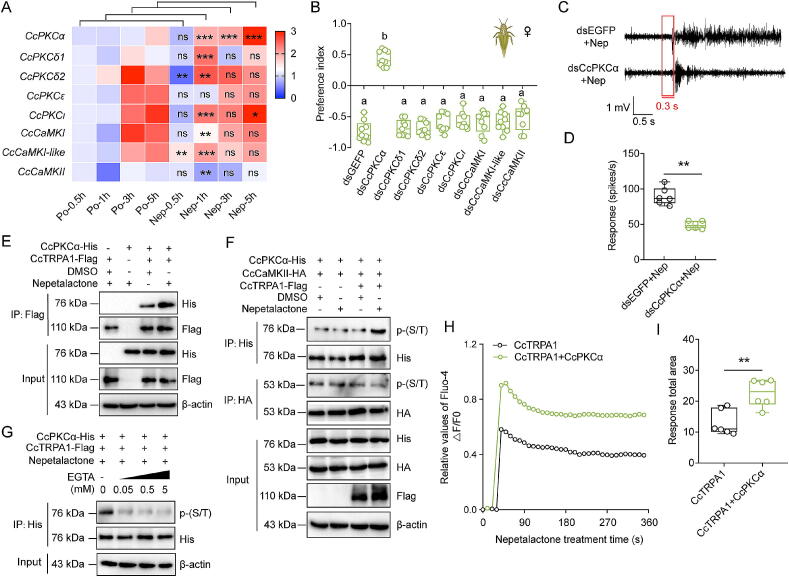
**CcPKCα interacts with CcTRPA1 to mediate nepetalactone-induced repellent behavior in *C. chinensis*.** A: Temporal expression profiles of PKC and CaMK homolog genes in *C. chinensis* after nepetalactone treatment (0.5, 1, 3, and 5 h). B: Behavioral impact of PKC/CaMK gene knockdown on nepetalactone-driven repellency in *C. chinensis* adults, assessed by dual-choice assays compared to dsEGFP treatment. C–D: Representative SSR traces and quantification of neural responses in CcTRPA1-expressing trichoid sensilla to 100 μM nepetalactone following *CcPKCα* silencing. E: Co-IP analysis confirming CcTRPA1-CcPKCα interaction. F-G: Nepetalactone-induced phosphorylation of CcPKCα bound to CcTRPA1, and its suppression by calcium chelation with EGTA. H-I: Calcium flux dynamics mediated by CcTRPA1-CcPKCα interaction upon nepetalactone stimulation. Data (A and B, D and I) are presented as mean ± SEM (n = 9 or 6; ≥30 insects/replicate). Significance: **p < 0.01, ***p < 0.001 (Student’s *t*-test); n.s.: no significant (*p* > 0.05). Letters denote significance (ANOVA, Tukey’s HSD; *p* < 0.05).

To dissect the downstream signaling mechanism underlying TRPA1-mediated repellency, we focused on calcium-dependent kinases—PKC and CaMK—as putative mediators of nepetalactone responses in *C. chinensis*. Transcriptional profiling revealed selective upregulation of *CcPKCα* at 1, 3, and 5 h following nepetalactone treatment, while other PKC isoforms (*CcPKCδ1*, *CcPKCδ2*, *CcPKCε*, *CcPKCι*) and CaMK family members (*CaCaMKI*, *CaCaMKI-like*, and *CaCaMKII*) remained unaffected at most timepoints (Fig. 4A). Genetic and pharmacological suppression of *CcTRPA1* (via RNAi or inhibitors AP18 and HC030031 treatments) abolished the mRNA expression of *CcPKCα*, but an effect fully rescued by nepetalactone co-treatment (Fig. S8A–B). Tissue-specific profiling showed that *CcPKCα* expressed higher in head compared to other 6 tissues, which is similar with *CcTRPA1* (Fig. S9A). Functional validation demonstrated *CcPKCα's* necessity for repellency: RNAi-mediated silencing (42.92 % knockdown efficiency) eliminated nepetalactone aversion (PI shift: −0.74 vs +0.44 dsEGFP controls) in *C. chinensis*, while suppression of other kinases had no effect (Figs. 4B and S9B). SSR results corroborated these findings, showing 45.35 % reduced neuronal firing rates in *CcPKCα*-silenced individuals (Fig. 4C–D). Mechanistically, Co-IP assays confirmed nepetalactone-dependent physical interaction between CcTRPA1 and CcPKCα in heterologous cells (Fig. 4E). Western blotting revealed rapid CcPKCα phosphorylation that was calcium-dependent (EGTA abolished activation) (Fig. 4F–G). Calcium imaging in co-transfected HEK293T cells demonstrated synergistic Ca^2+^ flux (Fluo-4 signal), confirming functional coupling (Fig. 4H–I). This multilevel analysis establishes CcPKCα as the critical downstream effector of CcTRPA1-mediated nepetalactone repellency, revealing an insect-specific signaling paradigm distinct from vertebrate TRPA1 pathways.

### Nepetalactone activates TRPA1 homologues to attract two predatory ladybeetles

**Fig. 5:**
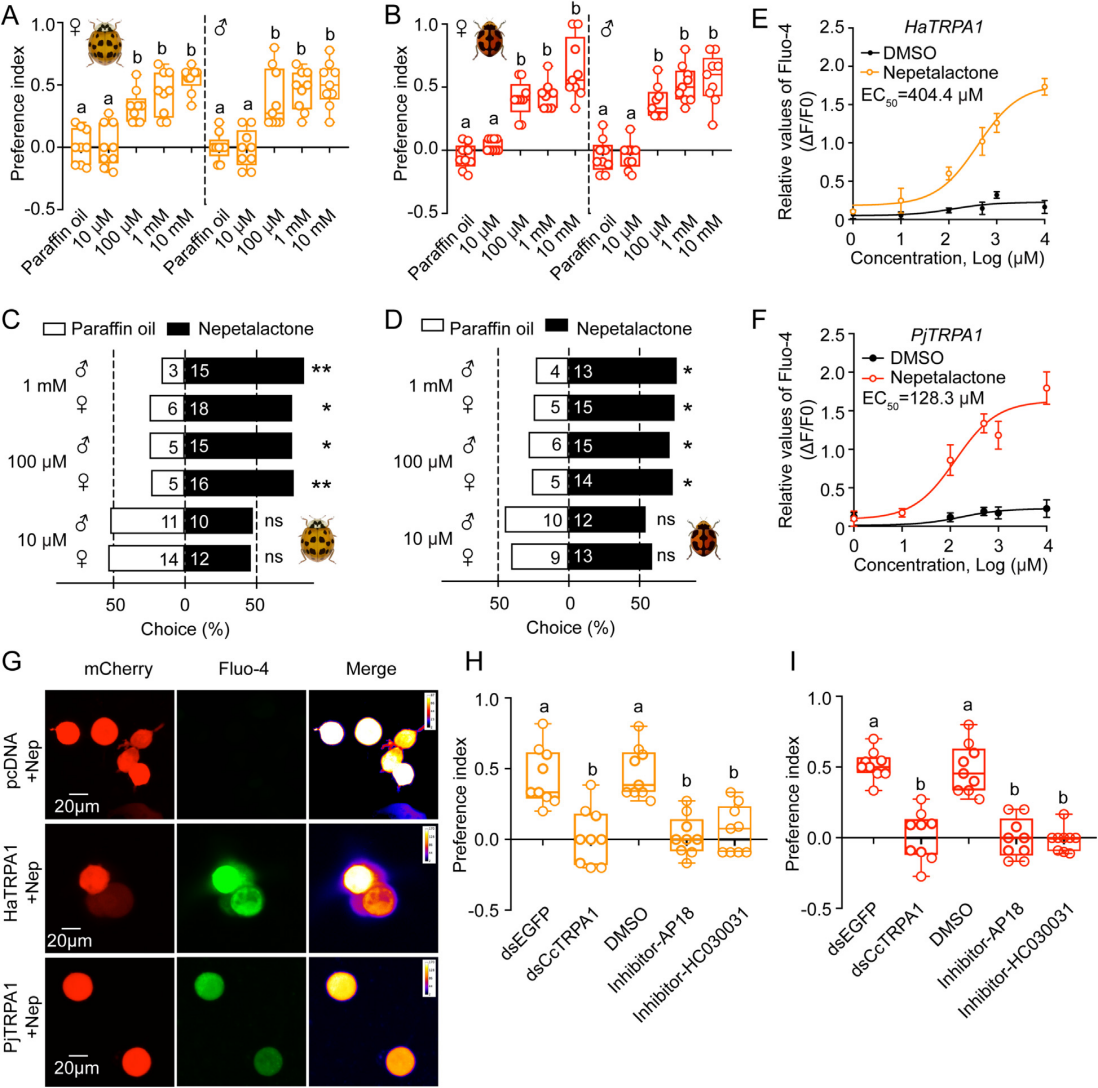
**TRPA1 activation by nepetalactone mediates attraction in two predatory ladybeetles.** A–B: Dose-dependent attraction of *H. axyridis* or *P. japonica* adults to nepetalactone (10 μM–10 mM) vs. paraffin oil controls in two-way choice assays. Data are indicated as mean values ± SEM from nine independent biological replicates, each consisting of approximately 12 adults of *H. axyridis* or *P. japonica*. The preference index was calculated as description above. C–D: Y-shaped tube olfactometer validation of nepetalactone’s attraction efficacy. Each group consisted of three independent biological replicates with a total of approximate 32 adults. E-F: Concentration-response curves of nepetalactone-induced calcium signaling in HEK293T cells heterologously expressing *HaTRPA1* or *PjTRPA1*. Recombinant vectors were constructed by cloning full-length *HaTRPA1*/*PjTRPA1* into pcDNA3.1(+)-mCherry. DMSO served as negative control. EC_50_ values drived from fitted curve. G: Representative Fluo-4 Ca^2+^ imaging of TRPA1-expressing HEK293T cells treated with nepetalactone. mCherry fluorescence (red) indicates transfected cells; Fluo-4 (green) shows intracellular Ca^2+^ levels. Scale bar is 20 μm. H-I: Attenuation of nepetalactone attraction by TRPA1 knockdown or pharmacological inhibition. **Data presentation**: Mean ± SEM (Panels A–B, E–F, H–I; n = 9 biological replicates; ∼12–32 insects/group). **Statistics**: Different lowercase letters indicate significant differences (ANOVA with Turkey’s test; *p* < 0.05). Chi-square test for panels C–D (ns: not significant; *p < 0.05; **p < 0.01). (For interpretation of the references to colour in this figure legend, the reader is referred to the web version of this article.)

The convergent evolution of TRPA1 signaling pathways across taxa raises a critical question: Can this receptor mediate opposing ecological behaviors in phylogenetically distinct species? We investigated this paradox using *H. axyridis* and *P. japonica* which are two generalist predators pivotal for biocontrol in agroecosystems [8,35,36]. Behavioral assays revealed concentration-dependent attraction to nepetalactone (100 μM–10 mM), with both species showing significant preference in two-way choice and Y-shaped tube assays (Fig. 5A–D). Strikingly, this response contrasts with the aversion observed in sap-sucking pests, suggesting divergent TRPA1 signaling outcomes. Functional characterization of ladybeetle TRPA1 orthologs (*HaTRPA1*, *PjTRPA1*) demonstrated nepetalactone-induced calcium flux in heterologous systems (Fig. 5E–G). The subcellular localization results indicated that HaTRPA1 and PjTRPA1 are mainly expressed on the cell membrane rather than the cytoplasm (Fig. S10). Moreover, RNAi-mediated *TRPA1* knockdown and pharmacological inhibitor abolished the attraction behavior of *H. axyridis* and *P. japonica* in response to nepetalactone compared to the control groups (Figs. S11A–D and 5H–I). These results unequivocally establish TRPA1 as the molecular mediator of nepetalactone attraction in predatory ladybeetles.

### HaTRPA1-HaCaMKII axis mediates nepetalactone-induced attraction in *H. axyridis*

**Fig. 6:**
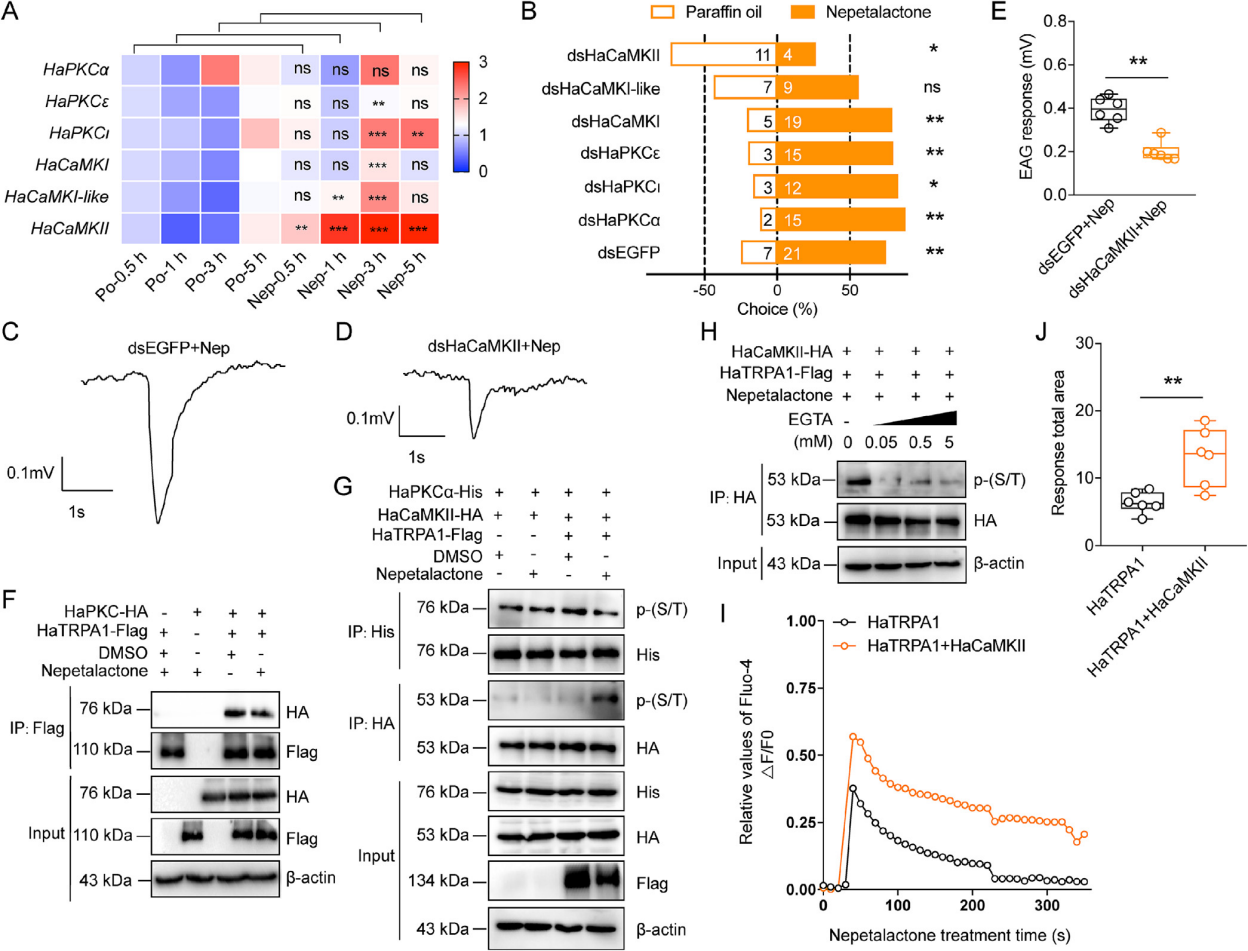
**HaCaMKII-HaTRPA1 interaction underlies nepetalactone-induced attraction in *H. axyridis*.** A: Time-course expression of PKC/CaMK homologs in *H. axyridis* after nepetalactone exposure (0.5, 1, 3, and 5 h). B: Behavioral effects of PKC/CaMK knockdown on attraction responses of nepetalactone in *H. axyridis* using Y-shaped tube assays compared to dsEGFP treatment. C–E: Representative EAG responses of HaTRPA1-expressing trichoid sensilla to nepetalactone after *HaCaMKII* silencing. F: Co-IP evidence of HaTRPA1-HaCaMKII interaction. G–H: Nepetalactone-induced phosphorylation of HaCaMKII bound to HaTRPA1 and EGTA-mediated suppression. I–J: Calcium flux dynamics regulated by HaTRPA1-HaCaMKII interaction. Data (A, E, and J) are presented as mean ± SEM (n = 9 or 6; ≥30 insects/replicate). Significance: **p < 0.01, ***p < 0.001 (Student’s *t*-test); n.s.: no significant (*p* > 0.05). Letters denote significance (ANOVA, Tukey’s HSD; *p* < 0.05).

To resolve the signaling divergence underlying TRPA1-mediated behavioral polarity, we investigated calcium-dependent kinases in *Harmonia axyridis*, identifying three PKC (*HaPKCα*, *HaPKCε*, and *HaPKCι*) and three CaMK (*HaCaMKI*, *HaCaMKI-like*, and *HaCaMKII*) isoforms. Transcriptional profiling revealed *HaCaMKII* as the sole kinase upregulated by nepetalactone treatments (at 0.5, 1, 3, and 5 h), while other isoforms remained unaffected (Fig. 6A). *HaTRPA1* suppression via RNAi or pharmacological inhibitors (AP18 and HC030031) treatments abolished the mRNA expression of *HaCaMKII*, an effect fully rescued by nepetalactone co-treatment (Fig. S12A–B). Tissue-specific profiling revealed that *HaCaMKII* expressed higher in antenna than other 8 tissues (Fig. S13A). Functional studies demonstrated *HaCaMKII’*s necessity for attraction: RNAi silencing (51.38 % efficiency) eliminated nepetalactone preference (Choice: 26.67 % vs 75.00 % dsEGFP), whereas other kinase knockdowns had no effect (Figs. S13B and 6B). Electroantennography confirmed this behavioral deficit, showing reduced EAG responses (49.32 %) in *HaCaMKII*-silenced *H. axyridis* (Fig. 6C–E). Mechanistic analyses revealed that nepetalactone-dependent HaTRPA1-HaCaMKII interaction via co-immunoprecipitation in heterologous cells (Fig. 6F). Phosphorylation assays showed rapid HaCaMKII activation requiring calcium influx (EGTA abolished phosphorylation) (Fig. 6G–H). Calcium imaging in co-transfected HEK293T cells demonstrated synergistic Ca^2+^ flux (Fluo-4 signals) (Fig. 6I–J). This multimodal evidence establishes HaCaMKII as the critical downstream effector of HaTRPA1-mediated attraction, contrasting starkly with the PKC-dependent repellency observed in pests.

### Synergistic pest control through nepetalactone-mediated attraction of natural enemies

**Fig. 7:**
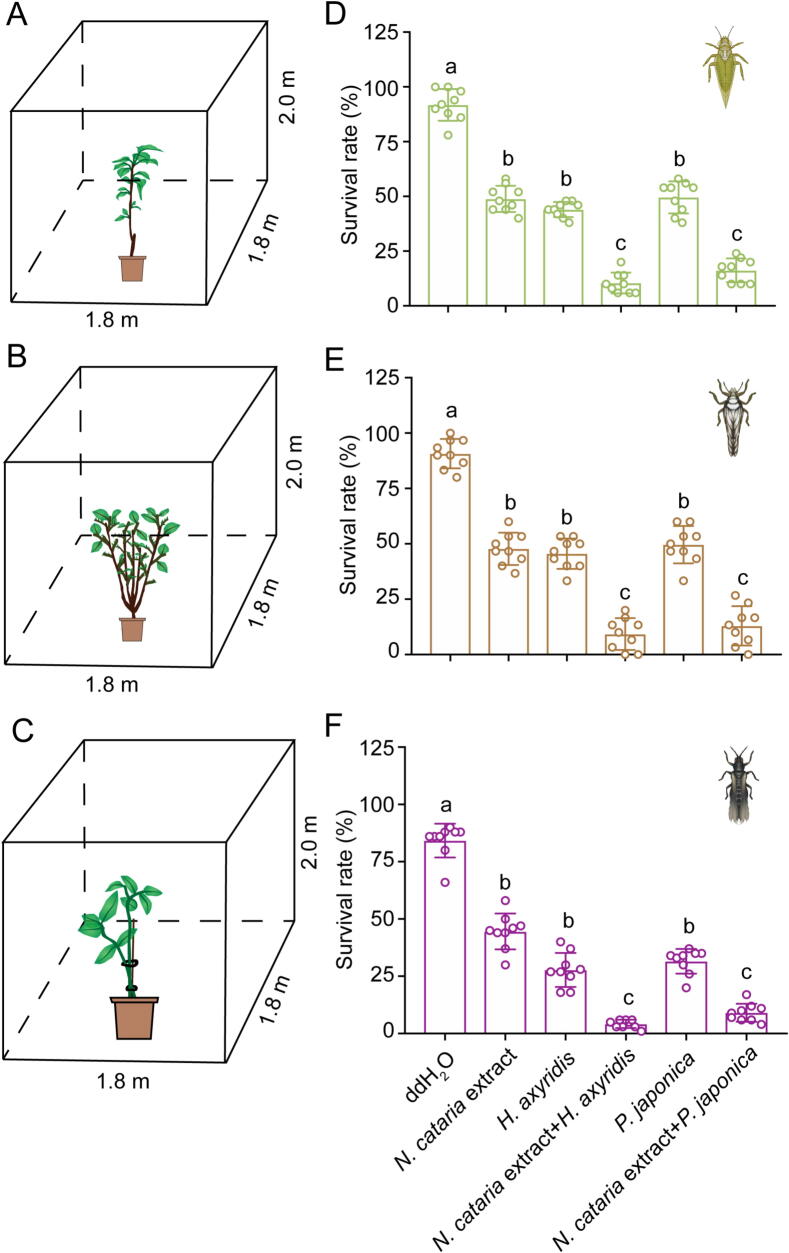
**Synergistic pest control by nepetalactone and predatory ladybeetles against Condylognatha pests.** A–C: Schematic of field cage assays evaluating nepetalactone-ladybeetles combinations against three Condylognatha pests: *C. chinensis*, *D. citri*, and *M. usitatus*. D–F: Pest survival rates 24 h post-treatment. *N. cataria* extract (5 mg/mL) was co-applied with 10 adults of *H. axyridis* or *P. japonica* per replicate. Data are shown as mean values ± SEM from nine independent biological replicates. Each replicate consisted of approximately 50 adults of *C. chinensis*, 30 adults of *D. citri*, or 100 adults of *M. usitatus*. Statistical significance was determined using ANOVA followed with Turkey’s multiple comparison test in SPSS 20.0 software. Different lowercase letters indicate significant differences (*p* < 0.05).

To operationalize the dual functionality of nepetalactone in repelling pests while recruiting predators, we evaluated its field efficacy within an integrated push–pull framework. Field cage experiments demonstrated stark contrasts in pest survival across treatments: control group (sterile water) exhibited 85 %–90 % survival rates for *C. chinensis*, *D. citri*, and *M. usitatus* (Fig. 7A–F). Individual applications of *N. cataria* extracts or predatory ladybeetles showed moderate efficacy. Strikingly, nepetalactone-ladybeetle synergy drove pest survival below 10 %, achieving near-complete population suppression. This combinatorial approach reduced chemical input costs by 60–75 % compared to conventional insecticide regimens while enhancing biocontrol persistence. Our findings validate nepetalactone as a keystone semiochemical for sustainable agriculture, bridging pest deterrence and predator recruitment through TRPA1-mediated ecological tuning.

## Discussion

The evolutionary proximity of Hemiptera and Thysanoptera within Condylognatha underscores their ecological significance as sap-sucking feeding pests with rapid reproduction cycles [37]. Current management of these species—exemplified by *C. chinensis*, *D. citri*, or *M. usitatus*—relies heavily on chemical insecticides [[38], [39], [40]], a strategy increasingly compromised by resistance evolution. Push-pull strategies, which exploit semiochemicals to manipulate pest behavior while minimizing environmental harm, offer a promising alternative [12,41,42]. However, their efficacy depends critically on identifying species-specific attractants/repellents tailored to ecological niches.

While nepetalactone has been established as a spatial repellent against dipteran and hemimetabolous pests [11,12,14,20,31], its role in holometabolous agricultural systems remained undefined. Our work bridges this gap by demonstrating nepetalactone’s dual functionality: (1) as a potent aversive agent for sap-sucking pests across nymphal and adult stages (Figs. 1 and S1), and (2) as a targeted attractant for key predatory beetles *H. axyridis* and *P. japonica* (Fig. 5). This bifurcated activity expands nepetalactone’s utility beyond traditional vector control, positioning it as a keystone semiochemical for sustainable agriculture. Notably, nepetalactone’s role as an aphid sex pheromone component and its ability to recruit generalist predators like *Chrysoperla carnea* suggest evolutionary co-option of TRPA1 signaling across trophic levels [43,44].

Indeed, gaining an understanding of the biological and chemical mechanism underlying the contrasting effects of nepetalactone on sap-sucking pests and predatory enemies can provide valuable guidance for the design and optimization of push–pull strategies [45,46]. Our findings mechanistically link opposing behavioral outcomes (repulsion vs*.* attraction) to divergent downstream kinases—PKC in pests versus CaMKII in predators (Fig. 4, Fig. 6). This kinase-dependent signaling polarity provides a molecular rationale for nepetalactone’s species-specific effects, addressing a critical knowledge gap in semiochemical ecology [40,47]. The conserved CVT binding motif in insect TRPA1 orthologs (vs. mammalian CPI variants) and functional validation through calcium imaging, mutagenesis, and chimeric assays (Figs. 2, S2–S4) confirm TRPA1 as the direct molecular target. The repellent effect in pests may arise from nepetalactone’s role as a constituent of alarm pheromones, while predators likely exploit it to locate prey—a dual evolutionary adaptation enhancing ecological regulation. Akin to the utilization of *Cnidium monnieri* in cotton cultivation [48], the cultivation of *N. cataria* directly in agricultural fields as a habitat management plant for its economic significance could aid in pest deterrence and conservation of natural enemies.

The operational synergy observed in field trials—where nepetalactone combined with predators achieved >90 % pest suppression (Fig. 7)—validates its practical value for integrated pest management. This aligns with emerging strategies leveraging semiochemicals like methyl salicylate and (E)-β-farnesene, which deter pests while recruiting predators [13,36,49]. By simultaneously exploiting TRPA1′s repulsive signaling in pests and attractive function in predators, this strategy amplifies ecological regulation while reducing insecticide dependence.

**Fig. 8:**
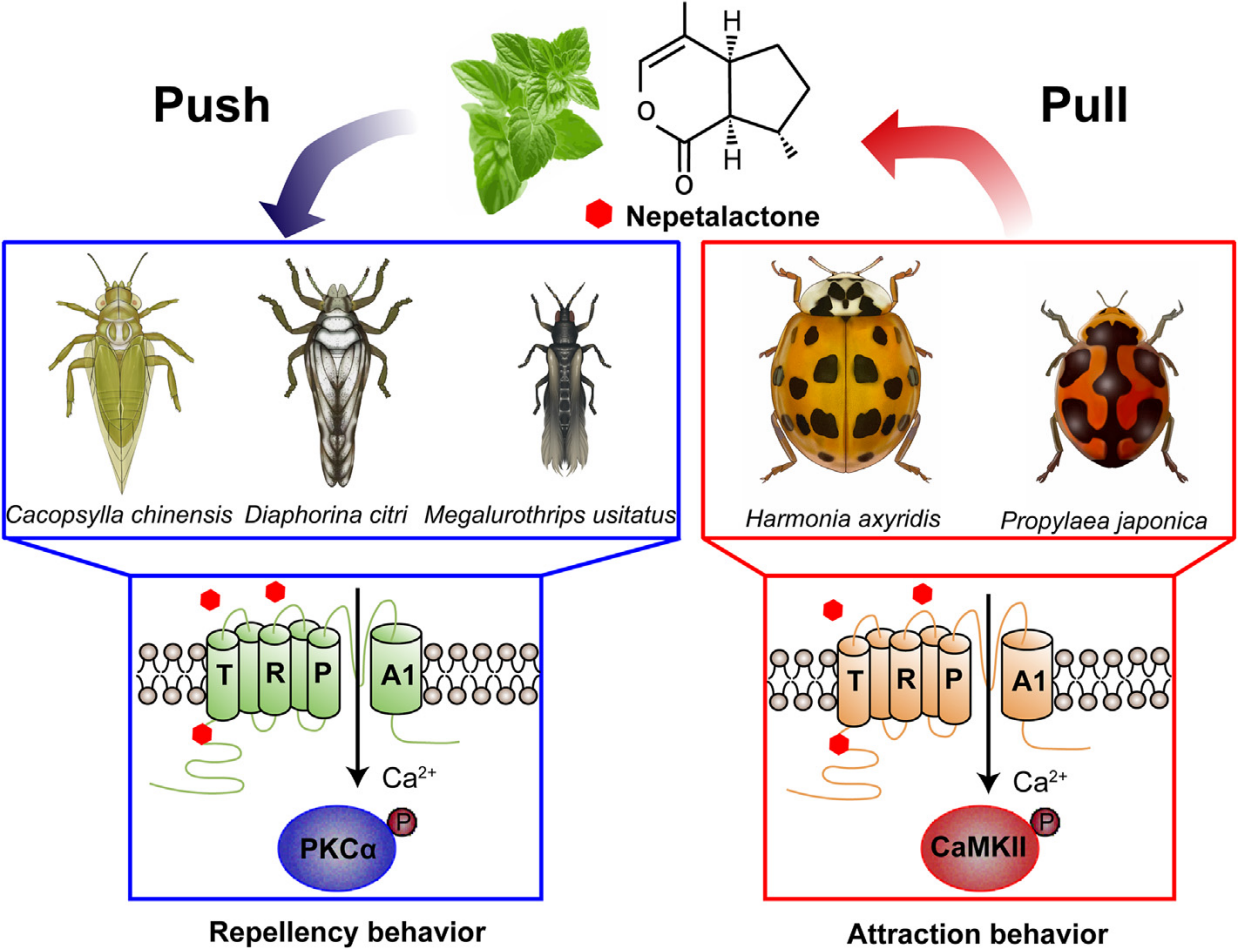
**Schematic representation of TRPA1-kinase divergence underlies nepetalactone’s dual ecological roles.** In pests (left), TRPA1-PKC signaling drives aversion. In predators (right), TRPA1-CaMKII activation mediates attraction.

In conclusion, our study reveals three key advances (Fig. 8): (1) **Behavioral specificity**: Condylognatha pests exhibit TRPA1-mediated aversion to nepetalactone, while predatory beetles display nepetalactone-dependent attraction; (2) **Mechanistic divergence**: PKCα and CaMKII drive opposing behavioral responses through conserved TRPA1 activation; (3) **Field efficacy**: Combining nepetalactone with natural enemies achieves near-complete pest suppression (**>90 %**). Moving forward, future research should prioritize: (1) **Ecological scalability**: Field trials across diverse crops (e.g., cotton, cereals) to assess environmental factors (wind, rainfall) influencing nepetalactone efficacy; (2) **Taxonomic expansion**: Investigating TRPA1-kinase interactions in Lepidoptera, Hymenoptera, and Orthoptera to broaden push–pull applicability; (3) **Synthesis and deployment**: Optimizing nepetalactone synthesis for cost-effective field use and exploring *Nepeta cataria* cultivation as a habitat management strategy. By resolving the TRPA1-kinase axis as a universal regulator of semiochemical responses, this work opens new avenues for precision biocontrol, harmonizing agricultural productivity with ecological sustainability.

## Compliance with ethics requirements

This article does not contain any studies with human subjects.

## Credit author statement

Conceived and designed the experiments: S.Z.; Performed the experiments: S.Z., J.L., B.W., and Y.W.; Analyzed the data: S.Z., J.L., Y.W., and B.W.; Contributed reagents/materials/analysis tools: F.L., Z.L., X.L.; Wrote and revised the paper: S.Z., and J.L.; Funding acquisition: S.Z.

## Declaration of competing interest

The authors declare that they have no known competing financial interests or personal relationships that could have appeared to influence the work reported in this paper.
