# Supplementary material for: TRPA1-kinase axis polarization: Nepetalactone drives pest repulsion and predator attraction via divergent PKC/CaMKII signaling
Source: J Adv Res. 2025 Jul 17;82:141–53. doi: 10.1016/j.jare.2025.07.026 (PMC13001051; doi:10.1016/j.jare.2025.07.026)
Supplement: Supplementary Data 1 [file mmc1.docx]

**Supplementary material**

**TRPA1-kinase axis polarization: Nepetalactone drives pest repulsion and predator attraction via divergent PKC/CaMKII signaling**

Jianying Li^1,2^, Bo Wang^2^, Yilin Wang^2^, Fen Li^3^, Zhen Li^2^, Xiaoxia Liu^2^, Songdou Zhang^1,2^*

^1^Plant Protection Research Institute, Guangdong Academy of Agricultural Sciences, Key Laboratory of Green Prevention and Control on Fruits and Vegetables in South China Ministry of Agriculture and Rural Affairs Guangdong Provincial Key Laboratory of High Technology for Plant Protection, Guangzhou 510640, P.R. China

^2^Department of Entomology and MOA Key Lab of Pest Monitoring and Green Management, College of Plant Protection, China Agricultural University, Beijing, 100193, China

^3^Sanya Nanfan Research Institute, Hainan University, Yazhou, Sanya 572024, China

*Correspondence: Songdou Zhang (S.Z.), zhangsongdou1128@126.com


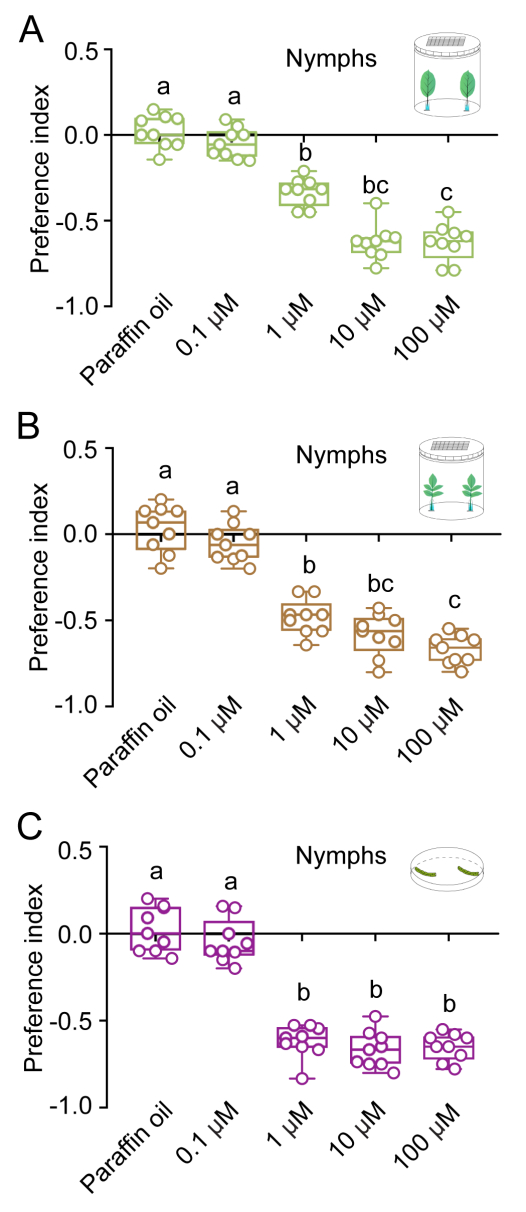


**Figure S1. Nymphs of Condylognatha pests exhibit heightened sensitivity to nepetalactone.**

A-C: Two-way choice assays for nymphs of *C. chinensis*, *D. citri*, and *M. usitatus*. Preference indexes (PI = [T-C]/[T+C]) for nepetalactone (0.1 to 100 μM) vs. paraffin oil control (mean values ± SEM, n = 9; 15-20 nymphs/replicate. Lowercase letters indicate significant differences (ANOVA, Turkey’s HSD; *p* < 0.05).


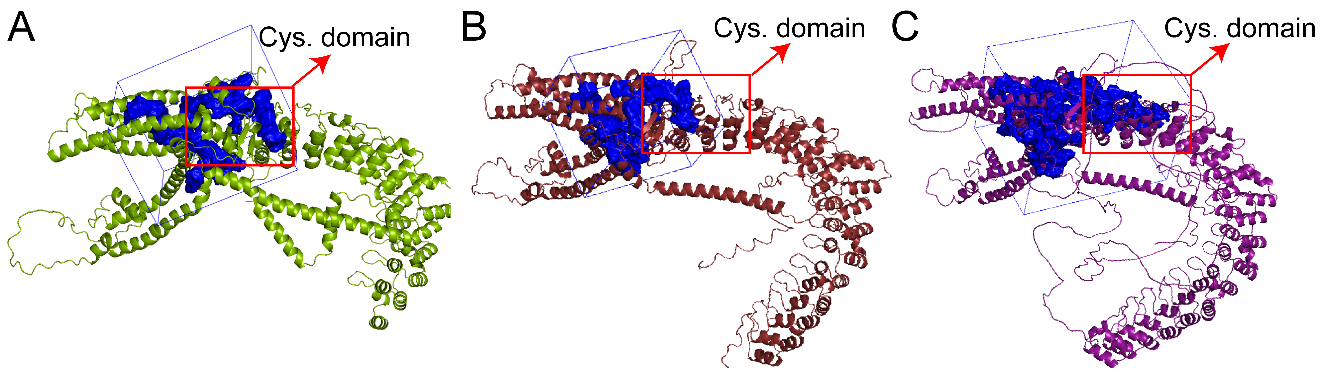


**Figure S2. Structural characterization of TRPA1 binding pockets.**

A-C: Surface representation of top-scoring docking pockets (blue) with cysteine-rich linker domains (red) in CcTRPA1, DcTRPA1, and MuTRPA1, respectively. Dashed squares highlight nepetalactone interaction regions.


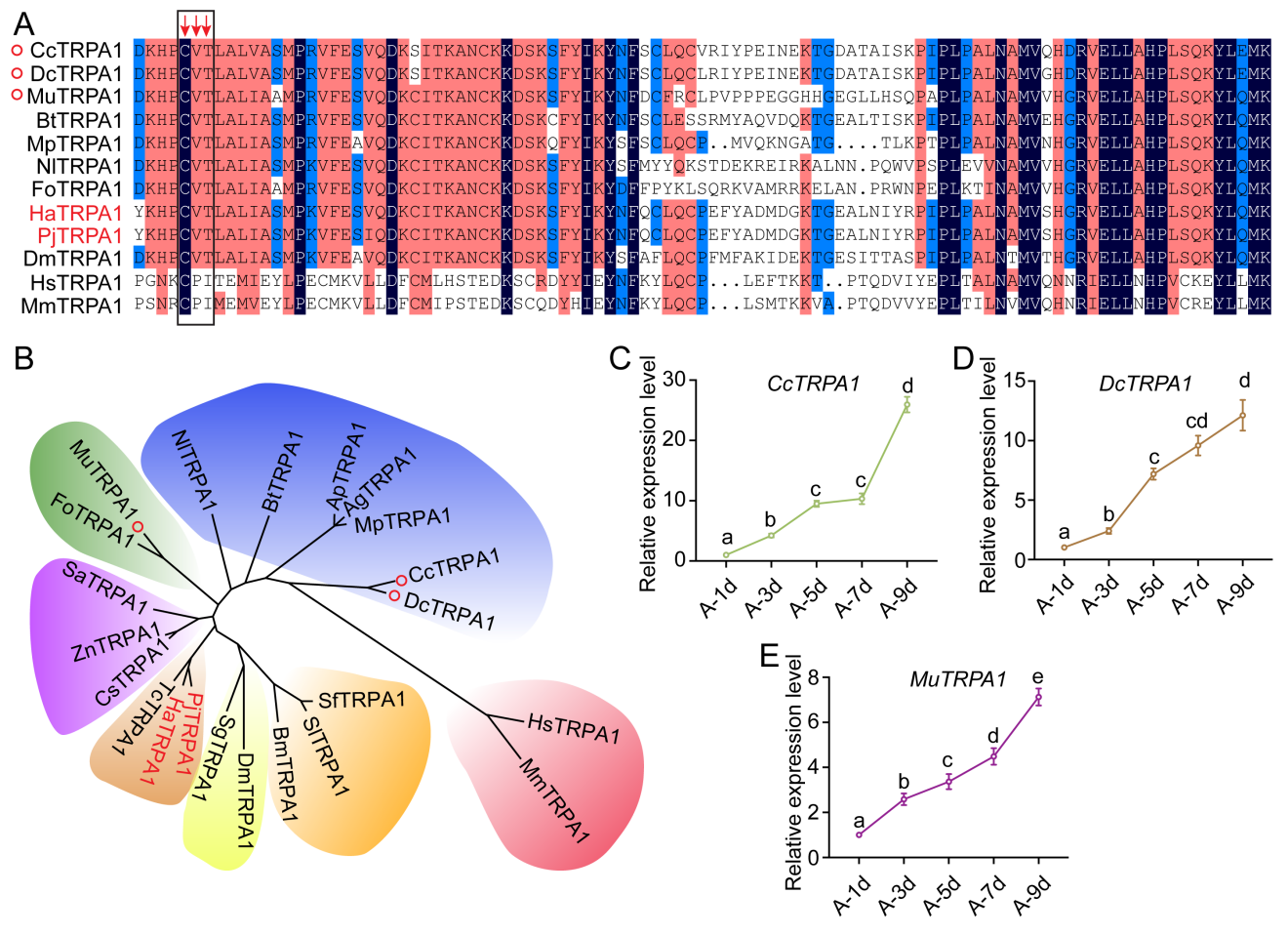


**Figure S3. Evolutionary and developmental dynamics of TRPA1 in Condylognatha.**

A: Multiple sequence alignment of TRPA1 orthologs (Clustal Omega). Black indicates 100% identity, red indicates 75% identity, and blue indicates identity below 75%. *CcTRPA1* (*Cacopsylla chinensis*, PP096834), *DcTRPA1* (*Diaphorina citri*, PP096835), *MuTRPA1* (*Megalurothrips usitatus*, PP096836), *BtTRPA1* (*Bemisia tabaci*, WMY99264.1), *MpTRPA1* (*Myzus persicae*, XP_022167295.1), *NlTRPA1* (*Nilaparvata lugens*, AOR81469.1), *FoTRPA1* (*Frankliniella occidentalis*, XP_052124278.1), *HarTRPA1* (*Helicoverpa armigera*, XP_049694764.1), *PjTRPA1* (*Propylaea japonica*, PP096837), *DmTRPA1* (*Drosophila melanogaster*, AEU17952.1), *HsTRPA1* (*Homo sapiens*, NP_015628.2), *MmTRPA1* (*Mus musculus*, NP_808449.1). Boxed residues denote nepetalactone binding sites. B: Phylogenetic analysis of TRPA1 homologs across arthropods and mammals. *AgTRPA1* (*Aphis gossypii*, XP_027848279.2), *ApTRPA1* (*Acyrthosiphon pisum*, XP_029342923.1), *SaTRPA1* (*Schistocerca americana*, XP_046988478.1), *ZnTRPA1* (*Zootermopsis nevadensis*, XP_021938934.1), *CsTRPA1* (*Cryptotermes secundus*, XP_023702415.1), *TcTRPA1* (*Tribolium castaneum*, XP_015834253.1), *SgTRPA1* (*Schistocerca gregaria*, XP_049832118.1), *BmTRPA1* (*Bombyx mori*, NP_001296525.1), *SlTRPA1* (*Spodoptera litura*, XP_022832910.1), *SfTRPA1* (*Spodoptera frugiperda*, XP_035438055.2). C: Developmental expression patterns of *TRPA1* in female adults. A-1d, A-3d, A-5d, A-7d, and A-9d represent female adults at 1, 3, 5, 7, and 9 days after eclosion, respectively.


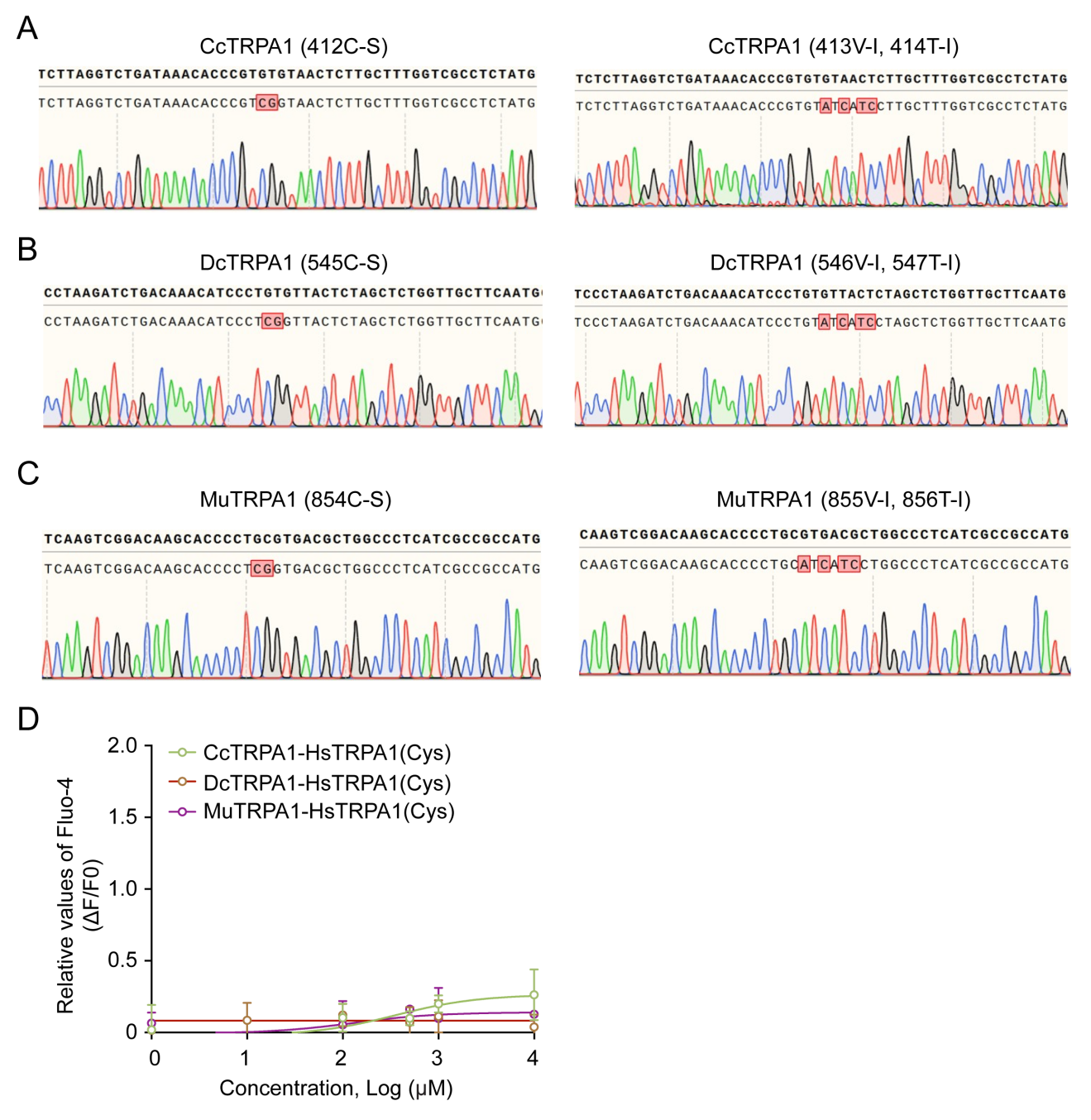


**Figure S4. Mutational validation and reciprocal domain-swap assays.**

A: Sequencing results for 412C-S, 413V-I, and 414T-I mutations in *CcTRPA1*. B: Sequencing results for 545C-S, 546V-I, 547V-I mutations in *DcTRPA1*. C: Sequencing results for 854C-S, 855V-I, 856T-I mutations in *MuTRPA1*. D: Inverse donor-recipient pair assays transferring related sites from humans to TRPA1 in the superorder Condylognatha.


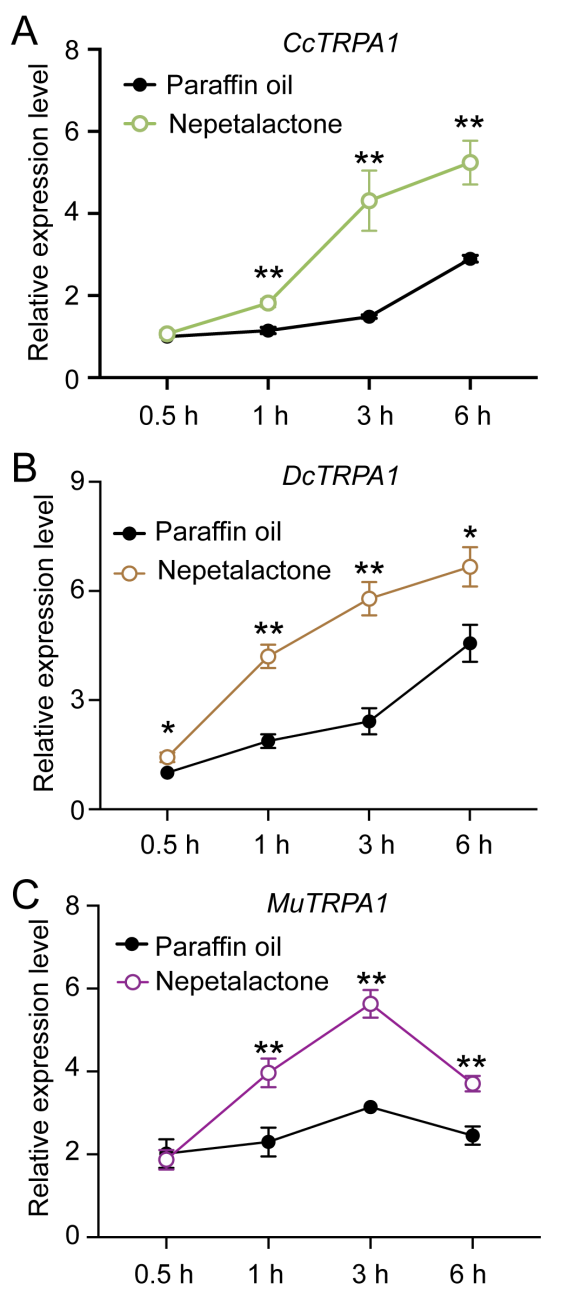


**Figure S5. Temporal regulation of *CcTRPA1*, *DcTRPA1*, and *MuTRPA1* expression by nepetalactone.**


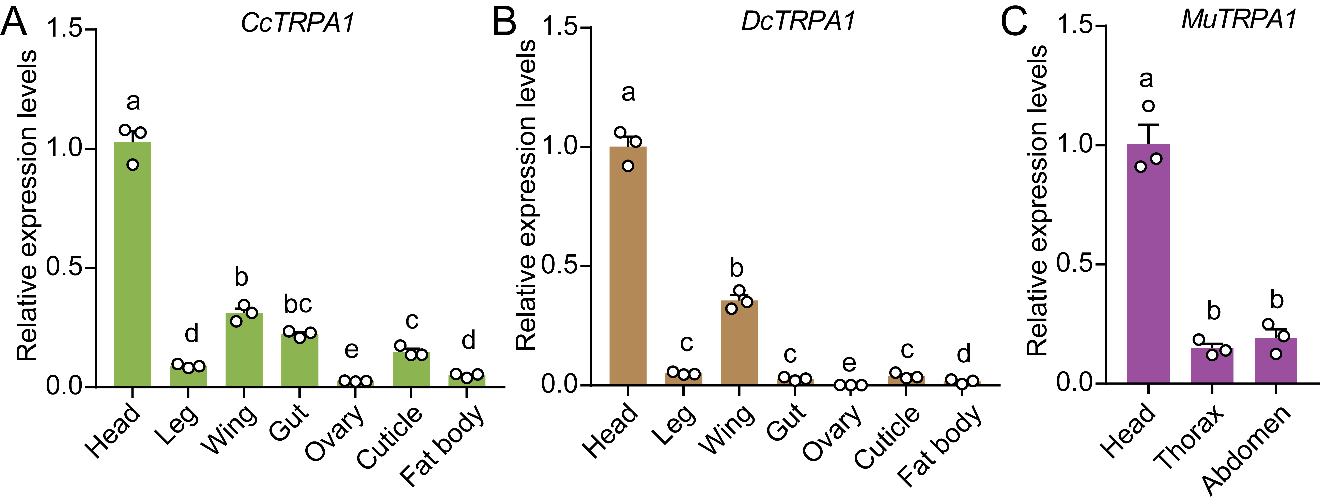


**Figure S6. Tissue-specific expression of *CcTRPA1*, *DcTRPA1*, and *MuTRPA1*.** Lowercase letters indicate significant differences (ANOVA, Turkey’s HSD; *p* < 0.05).


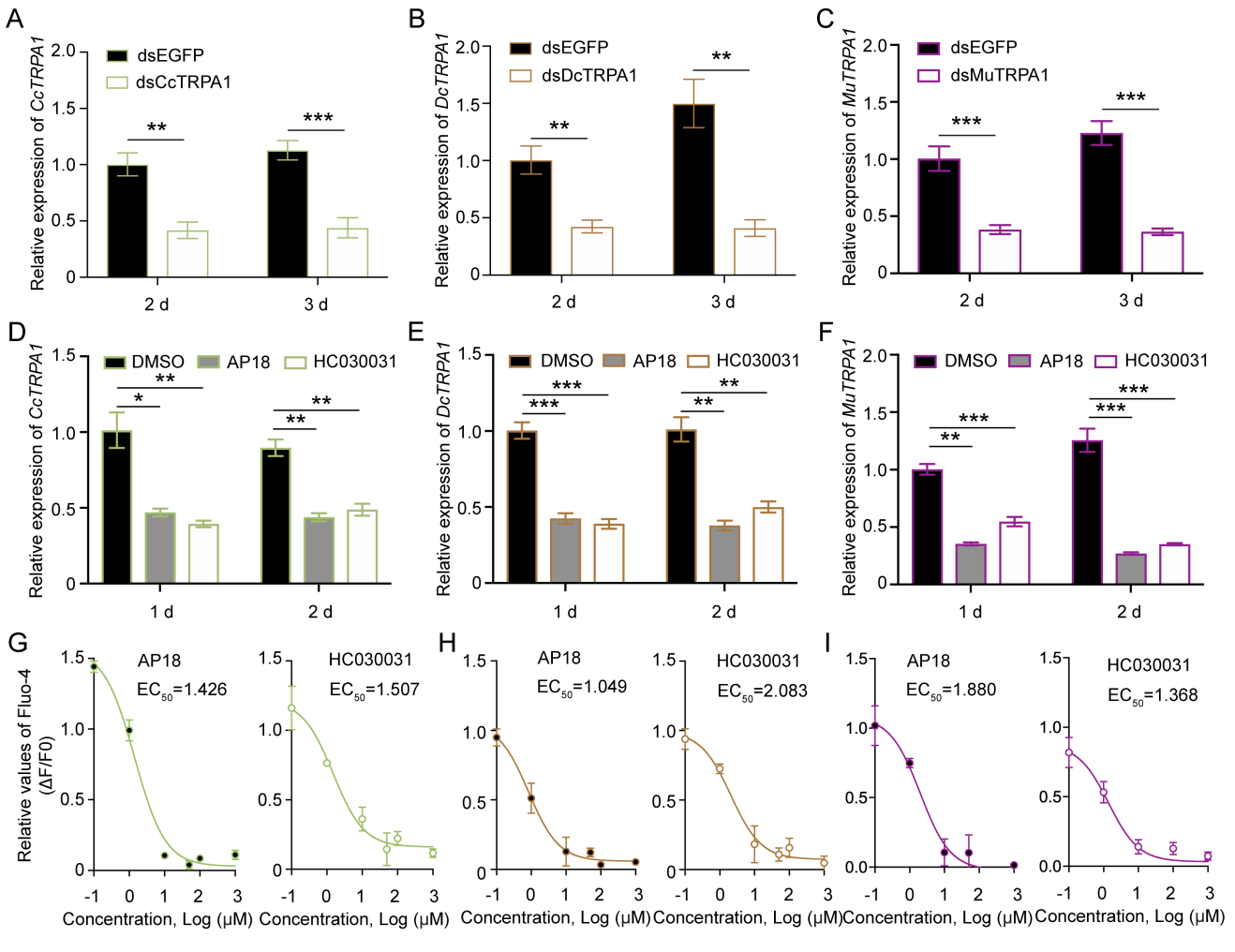


**Figure S7. TRPA1 suppression via RNAi and pharmacological inhibition.**

A-C: RNAi efficiency of *CcTRPA1*, *DcTRPA1*, and *MuTRPA1* at 2 and 3 days, respectively. D-F: Effect of of AP18 and HC030031 inhibitors on *TRPA1* mRNA expression at 1 and 2 days, separately. G-I: Dose-dependent calcium flux inhibition in TRPA1-expressing HEK293T cells after AP18 and HC030031 treatment.


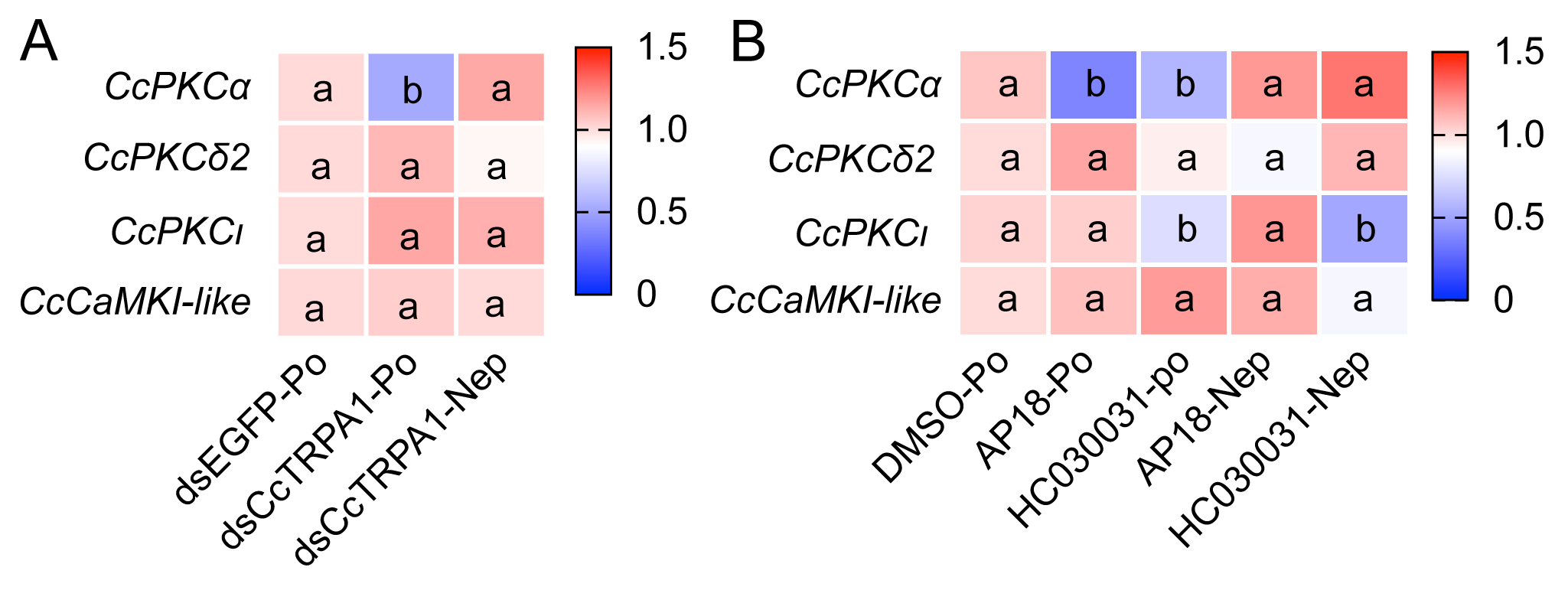


**Figure S8. Effect of *CcTRPA1* knockdown/inhibition on the mRNA expression of PKC and CaMK genes in *C. chinensis.***


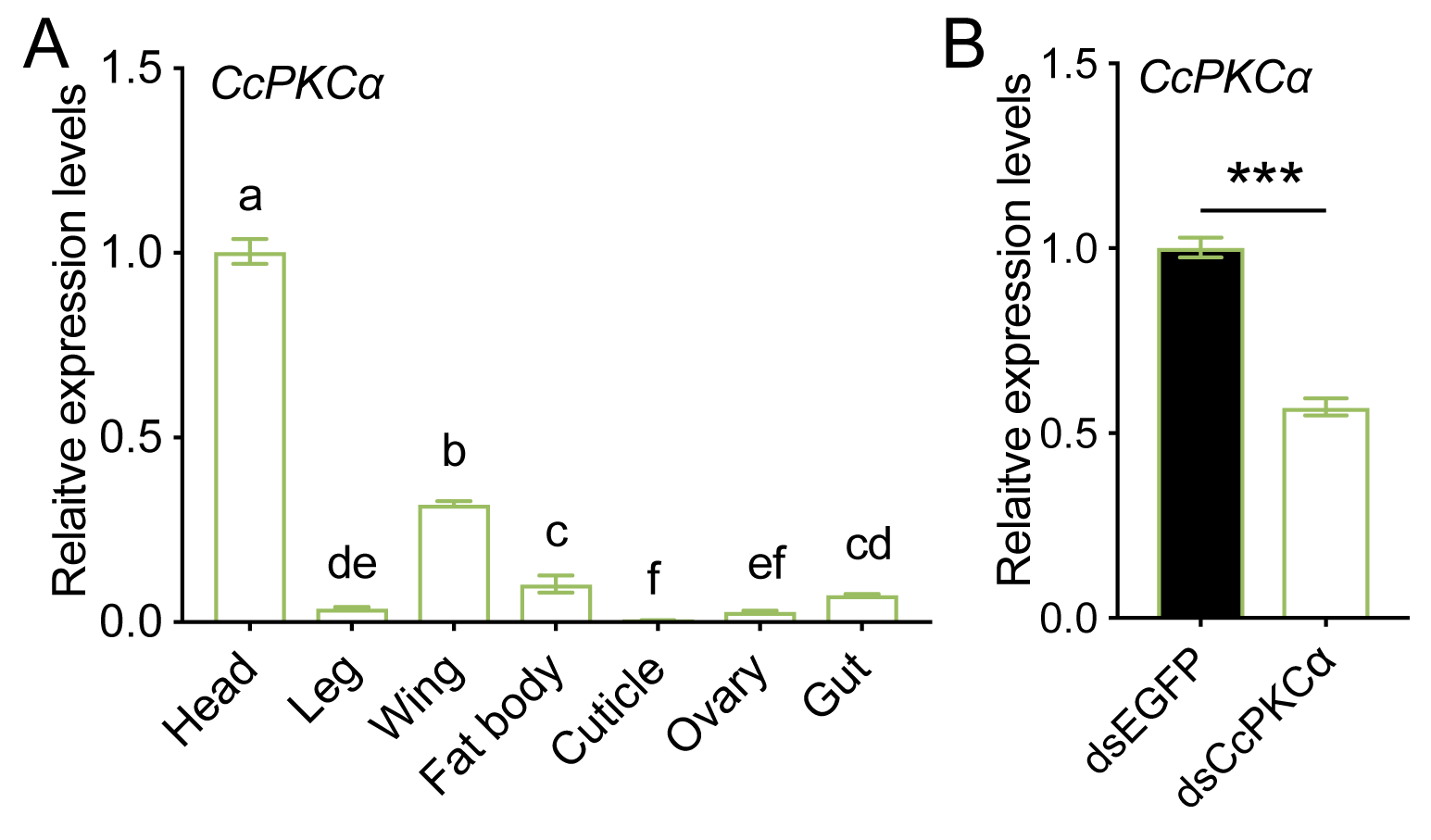


**Figure S9. Tissue-specific expression pattern and RNAi efficiency of *CcPKCα*.**


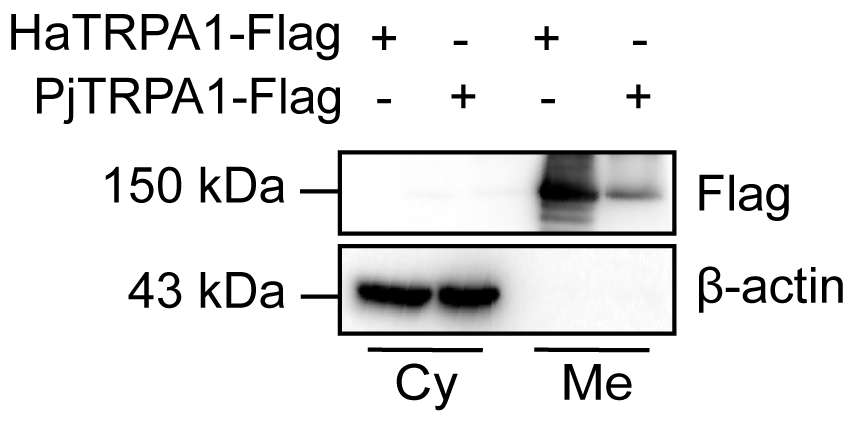


**Figure S10. Subcellular localization of HaTRPA1 and PjTRPA1.** Cy: Cytoplasm; Me: Membrane.


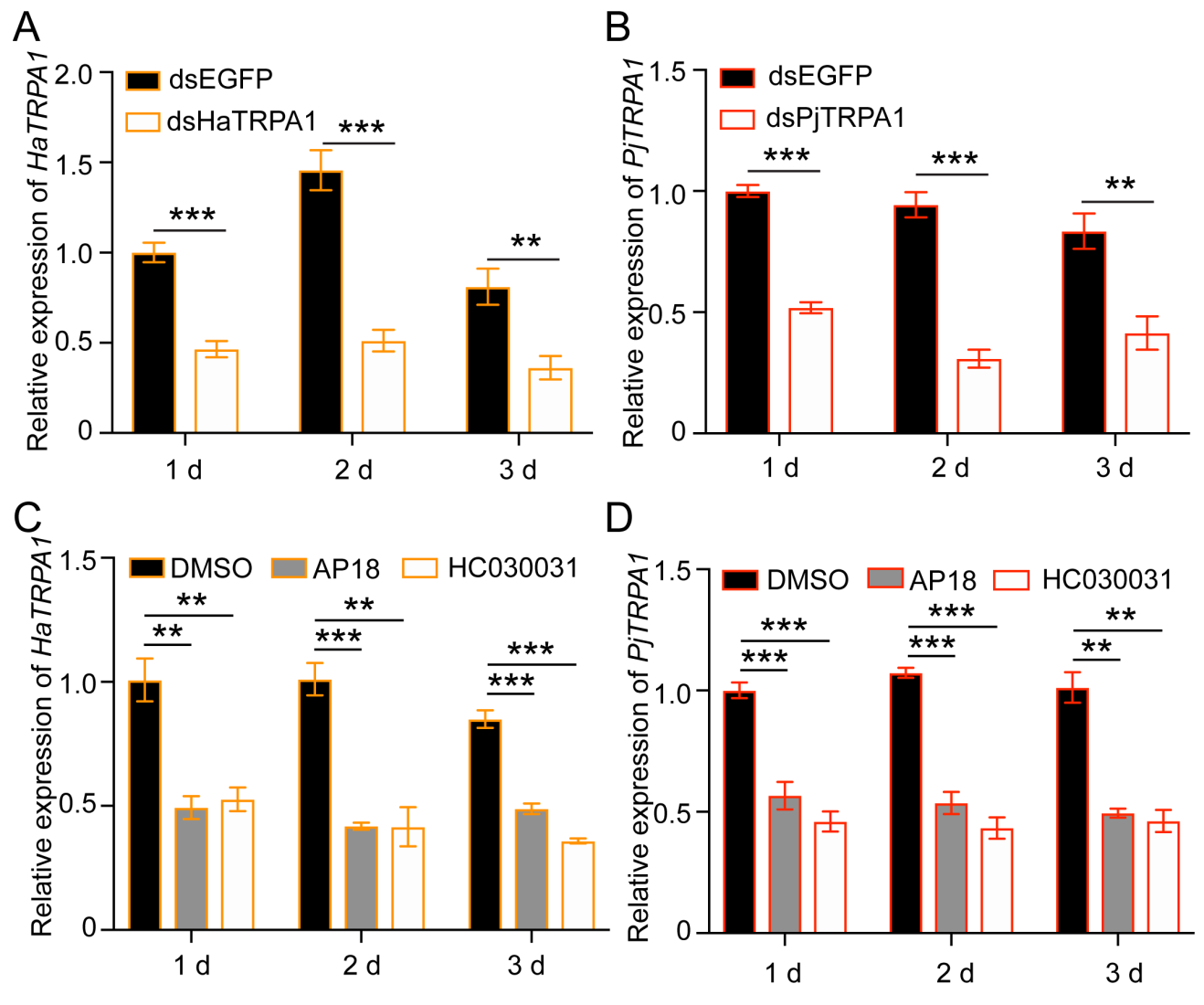


**Figure S11. RNAi and inhibitor treatments on the expression of *HaTRPA1* and *PjTRPA1*.**

A-B: Relative mRNA expression levels of *HaTRPA1* and *PjTRPA1* after dsRNA treatment on days 1, 2, and 3 compared to dsEGFP treatment, separately. C-D: Impact of inhibitor treatment on gene expression levels of *HaTRPA1* and *PjTRPA1* on days 1, 2, and 3 compared to DMSO treatment, respectively.


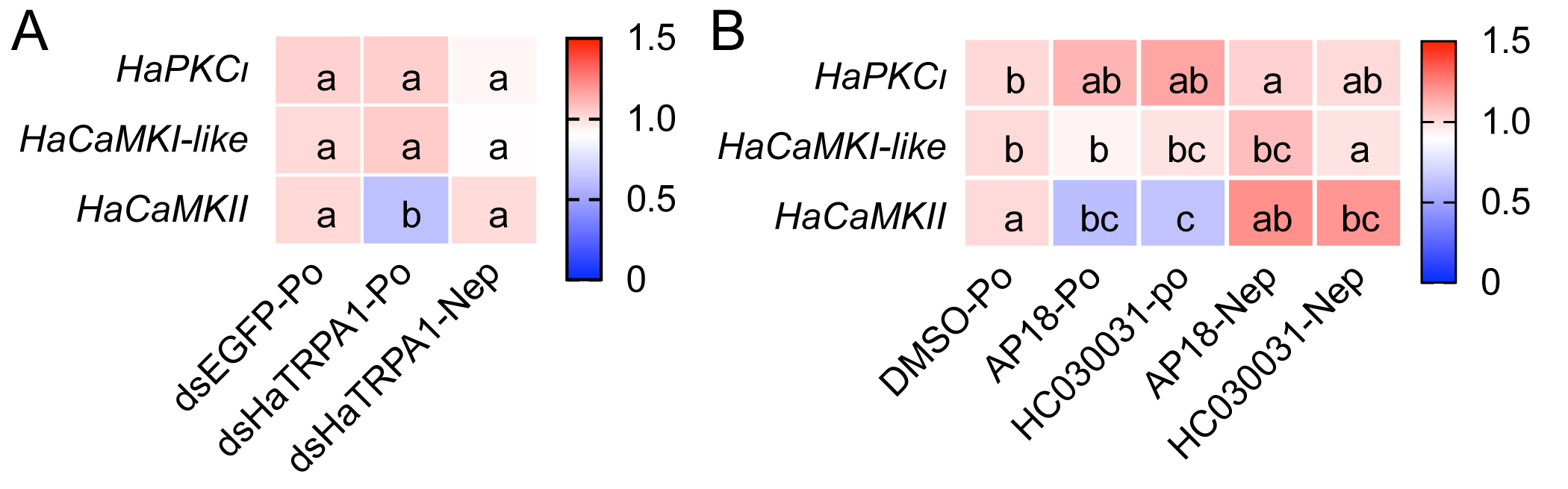


**Figure S12. Effect of *HaTRPA1* knockdown/inhibition on the mRNA expression of PKC and CaMK genes in *H. axyridis*.**

**
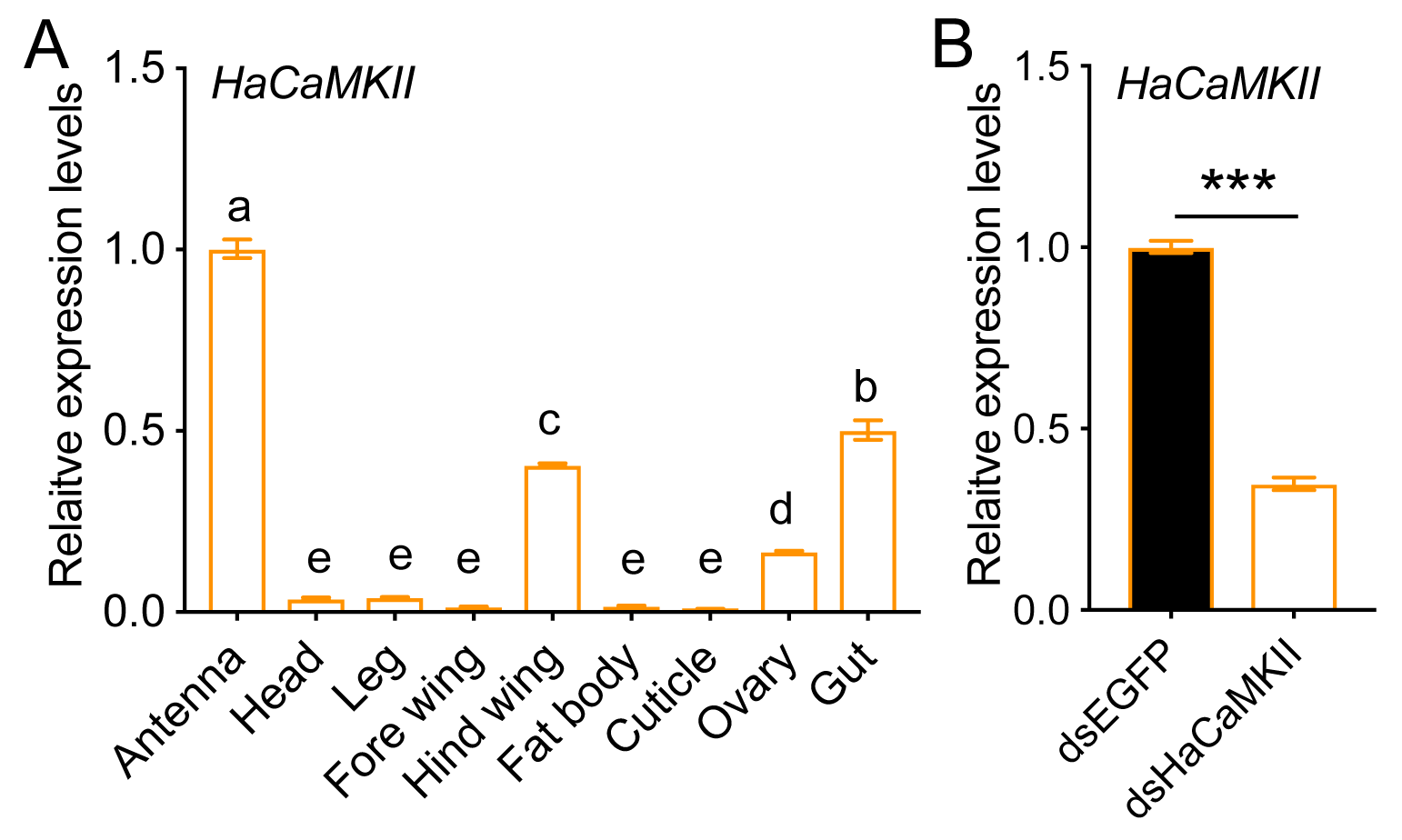
**

**Figure S13. Tissue-specific expression pattern and RNAi efficiency of *HaCaMKII*.**

**Table S1. The primer sequences used in current study.**

| **Gene name** | **Accession number** | **Sequences of primers (5′–3′)** | **Product length (bp)** | **Application purpose** |
| --- | --- | --- | --- | --- |
| *CcTRPA1* | PP096834 | Full-F: ATGGAGGTGTTTCTTCAGTA | 2859 | Full-length cDNA cloning |
|  |  | Full-R: CTAAAGAGACGACTTGGATT |  |  |
|  |  | qF: CTGATTGGTTTGGCTGTC | 150 | qPCR |
|  |  | qR: CTCGTAGATTTCCATTTTGTC |  |  |
|  |  | RNAi-F: GATCACTAATACGACTCACTATAGGGAGA  CCGAAATCATGTCTCTTAGG | 350 | dsRNA synthesis |
|  |  | RNAi-R: GATCACTAATACGACTCACTATAGGGAGA  GGATGTGCTAAGAGTTCGAC |  |  |
|  |  | orf-F: CCACTAGTCCAGTGTGGTGGAATTCG  ATGGAGGTGTTTCTTCAGTA | 2859 | Amplification of orf sequence |
|  |  | orf-R: CCTAGTCAGTCACTAGTGAT  CTAAAGAGACGACTTGGATT |  |  |
|  |  | CcTRPA1-Remove Cys -F:  TTCGGTTCCCCTTGTATTAT | 2541 | Amplification of TRPA1 chimeras’ sequence |
|  |  | CcTRPA1-Remove Cys -R:  AATGCCTATGGCAAATACTT |  |  |
|  |  | HsTRPA1-CcTRPA1-cys-F: CAGGAGCAAAAGATGGGATGAATGT  ATCATGTCTCTTAGGTCTGA | 318 |  |
|  |  | HsTRPA1-CcTRPA1-cys-R:  GAGCTCTAAATCCATAAGCCAA  CCATTTCATTTCCAGGTACT |  |  |
|  |  | CcTRPA1-C412S-F：  ATAAACACCCGTCGGTAACTCTTGCTTTGGTCGCCTCTAT | 2859 | Amplification of site-directed mutagenesis sequence |
|  |  | CcTRPA1-C412S-R：  AGTTACCGACGGGTGTTTATCAGACCTAAGAGACATGATT |  |  |
|  |  | CcTRPA1-V413I T414I-F：  ATAAACACCCGTGTATCATCCTTGCTTTGGTCGCCTCTAT |  |  |
|  |  | CcTRPA1-V413I T414I-R：  GATGATACACGGGTGTTTATCAGACCTAAGAGACATGAT |  |  |
|  |  | Flag-F: CCACTAGTCCAGTGTGGTGGAATTCGGCCACC  ATGGAGGTGTTTCTTCAGTA | 2856 | Western Blot |
|  |  | Flag-R: GTTTAAACGGGCCCTCTAGA  CTACTTGTCATCGTCATCCTTGTAGTCGATGTCATGATCTTTATAATCACCGTCATGGTCTTTGTAGTCCTCGAGAAGAGACGACTTGGATT |  |  |
| *DcTRPA1* | PP096835 | Full-F: ATGTCCACAGATCTCAACAT | 3222 | Full-length cDNA cloning |
|  |  | Full-R: TCATAGGGATGACTTGGATT |  |  |
|  |  | qF: AGAGTAATGGGAACGATGTG | 135 | qPCR |
|  |  | qR: TTTCGGTTTTGATATCCATC |  |  |
|  |  | RNAi-F: GATCACTAATACGACTCACTATAGGGAGA  ACTGCAAGAAAGACTCGAAG | 377 | dsRNA synthesis |
|  |  | RNAi-R: GATCACTAATACGACTCACTATAGGGAGA  GCCACAGATGTAGAGGAGAG |  |  |
|  |  | orf-F: CCACTAGTCCAGTGTGGTGGAATTCG  ATGTCCACAGATCTCAACAT | 123 | Amplification of orf sequence |
|  |  | orf-R: CCTAGTCAGTCACTAGTGAT  TCATAGGGATGACTTGGATT |  |  |
|  |  | DcTRPA1-Remove Cys -F:  CTCGGTGCCTCTTGTGTTGT | 2904 | Amplification of TRPA1 chimeras’ sequence |
|  |  | DcTRPA1-Remove Cys -R:  AATGCGTACGGCAAATACTT |  |  |
|  |  | HsTRPA1-DcTRPA1-cys-F: CAGGAGCAAAAGATGGGATGAATGT  ATCATGTCCCTAAGATCTGA | 318 |  |
|  |  | HsTRPA1-DcTRPA1-cys-R:  GAGCTCTAAATCCATAAGCCAA  CCACTTCATTTCCAAATATT |  |  |
|  |  | DcTRPA1-C545S-F：  ACAAACATCCCTCGGTTACTCTAGCTCTGGTTGCTTCAAT | 3222 | Amplification of site-directed mutagenesis sequence |
|  |  | DcTRPA1-C545S-R：  AGTAACCGAGGGATGTTTGTCAGATCTTAGGGACATGATC |  |  |
|  |  | DcTRPA1-V546I T547I-F：  ACAAACATCCCTGTATCATCCTAGCTCTGGTTGCTTCAAT | 3222 |  |
|  |  | DcTRPA1-V546I T547I-R：  GATGATACAGGGATGTTTGTCAGATCTTAGGGACATGATC |  |  |
|  |  | Flag-F: CCACTAGTCCAGTGTGGTGGAATTCGGCCACC  ATGTCCACAGATCTCAACAT | 3219 | Western Blot |
|  |  | Flag-R: ATCACCGTCATGGTCTTTGTAGTCCTCGAG TAGGGATGACTTGGATTTATTGGTC |  |  |
| *MuTRPA1* | PP096836 | Full-F: ATGCCCCACCGCCAGCCCGC | 4212 | Full-length cDNA cloning |
|  |  | Full-R: CTACGACTTGCTGTAGCTTC |  |  |
|  |  | qF: GTGAGGGTCTCCTCCACT | 114 | qPCR |
|  |  | qR: TCATCTGGAGGTACTTCTGC |  |  |
|  |  | RNAi-F: GATCACTAATACGACTCACTATAGGGAGA  TCCTTCATCATCAACGAGAG | 355 | dsRNA synthesis |
|  |  | RNAi-R: GATCACTAATACGACTCACTATAGGGAGA  AGTAGATGGCGTAGTCGATG |  |  |
|  |  | orf-F: CCACTAGTCCAGTGTGGTGGAATTCG  ATGCCCCACCGCCAGCCCGC | 4212 | Amplification of orf sequence |
|  |  | orf-R: CCTAGTCAGTCACTAGTGAT  CTACGACTTGCTGTAGCTTC |  |  |
|  |  | MuTRPA1-Remove Cys -F:  CACCTCCTGGGCCCTCTTGT | 3897 | Amplification of TRPA1 chimeras’ sequence |
|  |  | MuTRPA1-Remove Cys -R:  CACTCGTACGGCAAGTACTT |  |  |
|  |  | HsTRPA1-MuTRPA1-cys-F: CAGGAGCAAAAGATGGGATGAATGT  CTGGAGCTCAAGTCGGACAA | 315 |  |
|  |  | HsTRPA1-MuTRPA1-cys-R:  GAGCTCTAAATCCATAAGCCAA  CCACTTCATCTGGAGGTACT |  |  |
|  |  | MuTRPA1-C854S-F：  ACAAGCACCCCTCGGTGACGCTGGCCCTCATCGCCGC | 4212 | Amplification of site-directed mutagenesis sequence |
|  |  | MuTRPA1-C854S-R：  CGTCACCGAGGGGTGCTTGTCCGACTTGAGCTCCAGCACC |  |  |
|  |  | MuTRPA1-V855I T856I -F：  ACAAGCACCCCTGCATCATCCTGGCCCTCATCGCCGC |  |  |
|  |  | MuTRPA1-V855I T856I -R：  GATGATGCAGGGGTGCTTGTCCGACTTGAGCTCCAGCACC |  |  |
|  |  | Flag-F: CCACTAGTCCAGTGTGGTGGAATTCGGCCACC  ATGCCCCACCGCCAGCCCGC | 4209 | Western Blot |
|  |  | Flag-R: ATCACCGTCATGGTCTTTGTAGTCCTCGAG CGACTTGCTGTAGCTTCTCC |  |  |
| HaTRPA1 | XM_045607606.1 | Full-F: ATGAGATCAAAGATGCCTAC | 3831 | Full-length cDNA cloning |
|  |  | Full-R: TCATATGCTCTTATTGAAGT |  |  |
|  |  | qF: GTCCAGAAAATGGAGATCAA | 142 | qPCR |
|  |  | qR: GTCCAGAAAATGGAGATCAA |  |  |
|  |  | RNAi-F: GATCACTAATACGACTCACTATAGGGAGA  AACTCAGCGCGATAGACTAC | 386 | dsRNA synthesis |
|  |  | RNAi-R: GATCACTAATACGACTCACTATAGGGAGA  CTTTTGACTTAAGGGGTGTG |  |  |
|  |  | orf-F: CCACTAGTCCAGTGTGGTGGAATTCG  ATGAGATCAAAGATGCCTAC | 3831 | Amplification of orf sequence |
|  |  | orf-R: CCTAGTCAGTCACTAGTGAT  TCATATGCTCTTATTGAAGT |  |  |
|  |  | Flag-F: CCACTAGTCCAGTGTGGTGGAATTCGGCCACC  ATGAGATCAAAGATGCCTAC | 3828 | Western Blot |
|  |  | Flag-R: ATCACCGTCATGGTCTTTGTAGTCCTCGAG TATGCTCTTATTGAAGT |  |  |
| PjTRPA1 | PP096837 | Full-F: ATGATGGCCGAAAGTCCTTT | 3366 | Full-length cDNA cloning |
|  |  | Full-R: TCAAATACTTTTGTTGAAGT |  |  |
|  |  | qF: AACAATTTTTGCGACTCATT | 121 | qPCR |
|  |  | qR: GGTGATGTCCATTTATGTCC |  |  |
|  |  | RNAi-F: GATCACTAATACGACTCACTATAGGGAGA  GAATACCGCTCTACACTTGG | 356 | dsRNA synthesis |
|  |  | RNAi-R: GATCACTAATACGACTCACTATAGGGAGA  AGAATTCCGGACATTGTAAA |  |  |
|  |  | orf-F: CCACTAGTCCAGTGTGGTGGAATTCG  ATGATGGCCGAAAGTCCTTT | 3366 | Amplification of orf sequence |
|  |  | orf-R: CCTAGTCAGTCACTAGTGAT  ACTTCAACAAAAGTATTTGA |  |  |
|  |  | Flag-F: CCACTAGTCCAGTGTGGTGGAATTCGGCCACC  ATGATGGCCGAAAGTCCTTT | 3363 | Western Blot |
|  |  | Flag-R: ATCACCGTCATGGTCTTTGTAGTCCTCGAG AATACTTTTGTTGAAGTTTAGAACT |  |  |
| HsTRPA1 | NM_007332.3 | HsTRPA1-Remove Cys-F:  ACATTCATCCCATCTTTTGC | 3051 | Amplification of TRPA1 chimeras’ sequence |
|  |  | HsTRPA1-Remove Cys-R:  GCAAAAGATGGGATGAATGT |  |  |
|  |  | CcTRPA1-HsTRPA1-cys-F:  ATAATACAAGGGGAACCGAA  TTCAGTCATAATTCTCCAGG | 309 |  |
|  |  | CcTRPA1-HsTRPA1-cys-R:  TTACGGATACCGTTTATGAA  CCATTTCATGAGTAAATATTCTTT |  |  |
|  |  | DcTRPA1-HsTRPA1-cys-F:  ACAACACAAGAGGCACCGAG  TTCAGTCATAATTCTCCAGG |  |  |
|  |  | DcTRPA1-HsTRPA1-cys-R:  AAGTATTTGCCGTACGCATT  CCATTTCATGAGTAAATATTCTTT |  |  |
|  |  | MuTRPA1-HsTRPA1-cys-F:  ACAAGAGGGCCCAGGAGGTG  TTCAGTCATAATTCTCCAGG |  |  |
|  |  | MuTRPA1-HsTRPA1-cys-R:  AAGTACTTGCCGTACGAGTG  CCATTTCATGAGTAAATATTCTTT |  |  |
| *CcPKCα* | PQ818811 | Full-F: ATGGCCGACGAAGAGAATAGCAACGAA | 2007 | Full-length cDNA cloning |
|  |  | Full-R: GACATGTTGAATAAACTCTGGATTC |  |  |
|  |  | qF: TCCTCGAGAAGAAGAAGATG | 145 | qPCR |
|  |  | qR: GGTGAATTGTTTGTCGAAAT |  |  |
|  |  | RNAi-F: GATCACTAATACGACTCACTATAGGGAGA  TTCAATGTCAAGTGTGCAGT | 392 | dsRNA synthesis |
|  |  | RNAi-R: GATCACTAATACGACTCACTATAGGGAGA  GTACGGATCTGATTGACCAT |  |  |
|  |  | His-F: CCACTAGTCCAGTGTGGTGGAATTCGGCCACC  ATGGCCGACGAAGAGAATAGC | 2004 | Western Blot |
|  |  | His-R: GTTTAAACGGGCCCTCTAGA  CATGGTGATGGTGATGATGACCGGTACGCGTAGAATCGAGACCGAGGAGAGGGTTAGGGATAGGCTTACCGACATGTTGAATAAACTCTGGATTC |  |  |
| *CcPKCδ1* | PQ818812 | qF: CGACCATTGTGGATCTCTAT | 148 | qPCR |
|  |  | qR: AGAGGTTAACGCTTCTACGA |  |  |
|  |  | RNAi-F: GATCACTAATACGACTCACTATAGGGAGA  AAGTGGTTTCCAAAATGATG | 351 | dsRNA synthesis |
|  |  | RNAi-R: GATCACTAATACGACTCACTATAGGGAGA  TACAAAAAGCACAAAACGTG |  |  |
| *CcPKCδ2* | PQ818813 | qF: TTCAGTGGTTGTGATGAAGA | 132 | qPCR |
|  |  | qR: CAGTCTTTTGGTTGTGTCCT |  |  |
|  |  | RNAi-F: GATCACTAATACGACTCACTATAGGGAGA  GGTTGCTCCAGAGAGTACAG | 306 | dsRNA synthesis |
|  |  | RNAi-R: GATCACTAATACGACTCACTATAGGGAGA  GAGCTGGATGCATTATTTTC |  |  |
| *CcPKCε* | PQ818814 | qF: GTTACGTTAGCCTTGCAGTT | 124 | qPCR |
|  |  | qR: CCTCCTTACACATTCCAAAG |  |  |
|  |  | RNAi-F: GATCACTAATACGACTCACTATAGGGAGA  GTAGACGAAGACCATCTGGA | 372 | dsRNA synthesis |
|  |  | RNAi-R: GATCACTAATACGACTCACTATAGGGAGA  TCCGTTAATCTGGTGTATCC |  |  |
| *CcPKCι* | PQ818815 | qF: GTTGGAAGCAAAACAAGTTC | 145 | qPCR |
|  |  | qR: AGACTGGTCGATTTTCTCAA |  |  |
|  |  | RNAi-F: GATCACTAATACGACTCACTATAGGGAGA  GCCAAAAGATTCAATAGACG | 369 | dsRNA synthesis |
|  |  | RNAi-R: GATCACTAATACGACTCACTATAGGGAGA  TCCTCTTCCGATCACTCTAA |  |  |
| *CcCaMKI* | PV667786 | qF: GCATAGGCGTCATCTCTTAC | 130 | qPCR |
|  |  | qR: CTAATGTCGTCCCAATAAGG |  |  |
|  |  | RNAi-F: GATCACTAATACGACTCACTATAGGGAGA  GCACAATGTTTGCTGTAAAA | 370 | dsRNA synthesis |
|  |  | RNAi-R: GATCACTAATACGACTCACTATAGGGAGA  GGTTTTAAGTCTCGATGCAC |  |  |
| *CcCaMKI-like* | PV667787 | qF: GTGAGGACGATGTACGAAAT | 120 | qPCR |
|  |  | qR: AGTGACTTTCACCCAATGAG |  |  |
|  |  | RNAi-F: GATCACTAATACGACTCACTATAGGGAGA  GTCGTGTGAGTGTTGACAAG | 370 | dsRNA synthesis |
|  |  | RNAi-R: GATCACTAATACGACTCACTATAGGGAGA  CATACGGGTCATCAAGAACT |  |  |
| *CcCaMKII* | PV667788 | Full-F: ATGGCTGCCCCAGCAGCCTGC | 1698 | Full-length cDNA cloning |
|  |  | Full-R: TTACTTGTGTCCCAAAAGAG |  |  |
|  |  | qF: CATGGAGTTCCACAAGTTTT | 120 | qPCR |
|  |  | qR: TGCTTGTCCATGTATTGTGT |  |  |
|  |  | RNAi-F: GATCACTAATACGACTCACTATAGGGAGA  CATCGTTCACAGACAAGAGA | 390 | dsRNA synthesis |
|  |  | RNAi-R: GATCACTAATACGACTCACTATAGGGAGA  GACGTGGTAGTTGTGGTCTT |  |  |
|  |  | HA-F: CCACTAGTCCAGTGTGGTGGAATTCGGCCACC  ATGGCTGCCCCAGCAGCCTG | 1695 | Western Blot |
|  |  | HA-R: GTTTAAACGGGCCCTCTAGA  CTACTGAGCAGCGTAATCTGGAACGTCATATGGATAGGACCCTGCATAGTCCGGGACGTCATAGGGATAGCCCGCATAGTCAGGAACATCGTATGGGTACTCGAGCTTGTGTCCCAAAAGAGAACTA |  |  |
| *HaPKCα* | XM_045609273.1 | Full-F: ATGGAGGACAATATGACCGAAGA | 2004 | Full-length cDNA cloning |
|  |  | Full-R: TTACACGTGCTGAACGAATTCGG |  |  |
|  |  | qF1: GTTTGCAAAGGATTTCTCAC | 144 | qPCR |
|  |  | qR: CTTGAATGGTGGTTGAACTT |  |  |
|  |  | RNAi-F1: GATCACTAATACGACTCACTATAGGGAGA  CTCAGATGAGGAAAACTTCG | 351 | dsRNA synthesis |
|  |  | RNAi-R: GATCACTAATACGACTCACTATAGGGAGA  TGACGTATTCCATCACAAAA |  |  |
|  |  | His-F: CCACTAGTCCAGTGTGGTGGAATTCGGCCACC ATGGAGGACAATATGACCGAAGA | 2001 | Western Blot |
|  |  | His-R: TACCTTCGAACCGCGGGCCCTCTAGA  CACGTGCTGAACGAATTCGG |  |  |
| *HaPKCε* | XM_045627638.1 | qF: AAAGATGCCGTCAACATACT | 135 | qPCR |
|  |  | qR: TAGAGCAACCCAGTCAATTT |  |  |
|  |  | RNAi-F: GATCACTAATACGACTCACTATAGGGAGA  TCTCGGTAAAGGAAGTTTTG | 350 | dsRNA synthesis |
|  |  | RNAi-R: GATCACTAATACGACTCACTATAGGGAGA  ATATAACGCCGTTCTTATGC |  |  |
| *HaPKCι* | XM_045622393.1 | qF: CACGAGGGTCATATCAAGTT | 134 | qPCR |
|  |  | qR: AAGCCGTAGTCTTCTCCTCT |  |  |
|  |  | RNAi-F: GATCACTAATACGACTCACTATAGGGAGA  GAGCCTGTTCTTAGAGACGA | 359 | dsRNA synthesis |
|  |  | RNAi-R: GATCACTAATACGACTCACTATAGGGAGA  GTCTGCACCCAGTCTATGTC |  |  |
| *HaCaMKI* | XM_045611237.1 | qF: CTGGACACTCTCATCCTCAT | 144 | qPCR |
|  |  | qR: GCTATACAGCCTTCTGTTGG |  |  |
|  |  | RNAi-F: GATCACTAATACGACTCACTATAGGGAGA  AGTTGCTCAAAAAGTCTGGA | 359 | dsRNA synthesis |
|  |  | RNAi-R: GATCACTAATACGACTCACTATAGGGAGA  TAAATTGGCTGGCTTTATGT |  |  |
| *HaCaMKI-like* | XM_045626772.1 | qF: GGCTATCCACCATTTTATGA | 150 | qPCR |
|  |  | qR: TCTTTCCACGTTTACACACA |  |  |
|  |  | RNAi-F: GATCACTAATACGACTCACTATAGGGAGA  ACTGGAGCATTCTCTGTTGT | 367 | dsRNA synthesis |
|  |  | RNAi-R: GATCACTAATACGACTCACTATAGGGAGA  AAAGGAGATTTTCAGGCTTC |  |  |
| *HaCaMKII* | XM_045609202.1 | Full-F: ATGTCGGTGCCTGTGGCTACTAC | 1575 | Full-length cDNA cloning |
|  |  | Full-R: CTATTTACTGAATCCAAATGC |  |  |
|  |  | qF: AAGATGCAGCTTGTATAGCC | 122 | qPCR |
|  |  | qR: ATTTTGCCATTTGTTGTCTT |  |  |
|  |  | RNAi-F: GATCACTAATACGACTCACTATAGGGAGA  TAGTCGGTTATCCACCATTC | 359 | dsRNA synthesis |
|  |  | RNAi-R: GATCACTAATACGACTCACTATAGGGAGA  TCCATCTCCTTTCTTTACGA |  |  |
|  |  | HA-F: CCACTAGTCCAGTGTGGTGGAATTCGGCCACC  ATGTCGGTGCCTGTGGCTAC | 1572 | Western Blot |
|  |  | HA-R: ATAGTCAGGAACATCGTATGGGTACTCGAG  TTTACTGAATCCAAATGCACTTC |  |  |
| *Ccβ-actin* | OQ658571 | qF: CGTATGCAGAAGGAAATCAC | 139 | qPCR |
|  |  | qR: AGATCCACATCTGTTGGAAG |  |  |
| *Dcβ-actin* | DQ675553.1 | qF: TGTTCCAACCTTCCTTCCTG | 109 | qPCR |
|  |  | qR: GTGTTGGCGTACAGGTCCTT |  |  |
| *Muβ-actin* | OK574306.1 | qF: GACCTCACCGACTACCTCAT | 105 | qPCR |
|  |  | qR: TAGCAGAGCTTCTCCTTGAC |  |  |
| *Haβ-actin* | MG983770.1 | qF: TGGGACAAAAGGACTCATAC | 144 | qPCR |
|  |  | qR: GGAGTTCGTTGTAGAAGGTG |  |  |
| *Pjβ-actin* | KJ522777.1 | qF: TTGGACTTCGAACAAGAAAT | 140 | qPCR |
|  |  | qR: AAGAAGGAAGGTTGGAAGAG |  |  |
| *EGFP* | ACY56286 | RNAi-F: GATCACTAATACGACTCACTATAGGGAGA ACTCCAGCAGGACCATGTGATC | 596 | dsRNA synthesis |
|  |  | RNAi-R GATCACTAATACGACTCACTATAGGGAGA  ACCTGAAGTTCATCTGCACCAC |  |  |

Note: The black boxes showed the T7 promoter sequences in the primers of dsRNA synthesis. The underline indicated the homologous arm sequence used for vector seamless clone. The dashed line represents the increased Tag protein sequence.
